# Systematic review and meta-analysis of the effectiveness of polypeptide, virus-like particles, and viral vector vaccines for foot-and-mouth disease (2020–2025)

**DOI:** 10.1038/s41598-025-24078-5

**Published:** 2025-11-10

**Authors:** Alyaa Elrashedy, Walid Mousa, Mohamed Nayel, Akram Salama, Ahmed Zaghawa, Ahmed Elsify, Mohamed E. Hasan

**Affiliations:** 1 Department of Animal Medicine and Infectious Diseases (Infectious Diseases), Faculty of Veterinary Medicine, University of Sadat City, Sadat City, Egypt; 2 Bioinformatics Department, Genetic Engineering and Biotechnology Research Institute, University of Sadat City, Sadat City, Egypt

**Keywords:** FMDV, Immune response, Peptide-based vaccine, VLP, Vector vaccine, Bibliometric analysis, Biotechnology, Diseases, Immunology, Microbiology

## Abstract

Foot-and-mouth disease (FMD) remains one of the most contagious viral diseases affecting livestock, posing major economic and food security challenges. This systematic review and meta-analysis evaluated recent advances in FMD vaccine strategies and their protective efficacy across platforms. The inclusion criteria were met by 24 out of 3,169 evaluated studies. Bibliometric trends highlighted growing interest in FMD vaccine technologies and the VP1 protein as a key immunogen. Innovative delivery methods, such as nanoliposomes, virus-like particles (VLPs), and dendrimeric peptides, have been linked to enhanced immune responses. A meta-analysis of studies conducted between 2020 and 2025 was performed to compare the protective effectiveness of four main vaccine platforms: peptide-based, viral vector, VLP, and dendritic cell-based vaccines. Subgroup analysis showed that VLP (RR = 1.66, 95% CI: 0.97–2.86) and viral vector vaccines (RR = 1.90, 95% CI: 0.08–46.65) demonstrated higher protection compared to other platforms, though wide confidence intervals suggest variability in efficacy across studies. Peptide vaccines demonstrated moderate efficacy (RR = 1.09, 95% CI: 0.75–1.57), whereas dendritic cell-based vaccines provided limited benefit. These findings support further development of peptide and VLP vaccines and underscore the role of advanced delivery systems in improving FMD vaccine effectiveness and contributing to One Health goals.

**Supplementary Information:**

The online version contains supplementary material available at 10.1038/s41598-025-24078-5.

## Introduction

Foot-and-mouth disease (FMD) is a highly contagious viral disease^1^. It spreads rapidly among cloven-hoofed animals, including cattle, pigs, sheep, and goats, leading to significant economic losses, involving production losses, trade restrictions, and costly control measures^2–4^. Given the global reliance on livestock for food security and economic stability, controlling FMD remains a top priority for the veterinary and agricultural sectors.

The virus responsible, foot-and-mouth disease virus (FMDV), belongs to the Picornaviridae family and is notorious for its genetic diversity, with seven distinct serotypes (O, A, C, Asia1, SAT1, SAT2, and SAT3) and numerous sublineages^5,6^. This diversity complicates vaccine development and disease control. For decades, inactivated whole-virus vaccines have been the cornerstone of FMD control programs^7^. These vaccines have been effective in reducing outbreaks, but they come with several limitations^8^. The production process requires large-scale virus cultivation under high biosafety conditions, raising concerns about accidental virus escape^9^. There is also a risk of incomplete virus inactivation, which could lead to unintended disease transmission^10,11^. Additionally, these vaccines demand stringent cold-chain storage and transportation, making them less practical for use in low-resource settings^12,13^. Due to FMDV’s antigenic diversity, these vaccines offer limited cross-protection and short-lived immunity, necessitating frequent booster doses and reformulations to address the virus’s rapid evolution^14–16^. Continuous surveillance in endemic regions is essential to keep pace with these changes. Additionally, while distinguishing infected animals from vaccinated ones (DIVA) was historically a challenge^17^. This issue has been addressed to some extent by the current inactivated FMDV vaccines. These vaccines, in combination with serological tests targeting non-structural proteins such as 3ABC, allow effective DIVA, as vaccinated animals do not typically produce antibodies against 3ABC, whereas infected animals do. This is critical for demonstrating freedom from disease in vaccinated populations and for effective surveillance and control^18,19^. These challenges have driven the search for safe, more effective, and broadly protective alternatives.

Emerging vaccine platforms also offer promising DIVA capabilities. Peptide-based vaccines, by design, exclude non-structural proteins (NSP), allowing straightforward differentiation when paired with NSP-targeted assays. Virus-like particles (VLPs) vaccines consist only of structural proteins, likewise preserving DIVA compatibility. Viral vector vaccines can be engineered to express specific antigens without NSPs, thereby enabling or even improving DIVA application compared to traditional inactivated vaccines^17,20^. Incorporating these features into next-generation vaccine strategies could strengthen surveillance programs while maintaining robust protection in endemic regions.

Among these, vaccines targeting the VP1 protein, critical for viral attachment, have emerged as a key immunogen^20–23^. Advances in epitope mapping and recombinant expression have reinforced its role as a leading vaccine target^24–29^.

Beyond antigen selection, vaccine delivery methods have also evolved. Traditional injectable vaccines are now being supplemented with novel delivery systems, such as nanoliposomes, VLPs, and oral vaccine formulations^30–33^. These innovations not only enhance immune response durability but also improve ease of administration, reducing the need for repeated booster doses. Such advancements could significantly benefit farmers in remote or resource-limited regions, where maintaining regular vaccination schedules can be challenging. Although small animal models like mice and guinea pigs are used for early immunogenicity studies, cattle and pigs remain the most relevant species for evaluating vaccine performance. Notably, pigs tend to be more difficult to immunize effectively than cattle using conventional vaccines, necessitating tailored approaches for each species^34^.

This review integrates systematic, meta-analytic, and bibliometric approaches to provide a comprehensive assessment of current FMDV vaccine research. The primary goal was to use meta-analysis to evaluate the comparative protective efficacy of peptide-based, VLP, viral vector, and dendritic cell–based vaccines across studies from 2020 to 2025. The secondary objective was to conduct a bibliometric analysis to contextualize meta-analytic findings, linking efficacy outcomes to global research trends, hotspots, and collaborations. Together, these findings aim to guide future vaccine development and inform strategies that align with the One Health framework, supporting livestock health, reducing antibiotic reliance, and protecting global food systems.

## Methods

### Literature search strategy

A comprehensive literature search was conducted across Web of Science Core Collection, Google Scholar, Scopus, PubMed, and ScienceDirect to identify relevant studies for FMDV vaccines. The search was restricted to peer-reviewed studies published between 2020 and 2025 to capture the most recent advanced developments in FMDV vaccines. This period was chosen to reflect the rise of novel design approaches, including bioinformatics-driven epitope prediction, VLP platforms, andinnovative delivery systems.

Boolean search queries were tailored for each database, using terms such as “foot-and-mouth disease” OR “FMDV” AND “vaccine” NOT “hand” to exclude unrelated topics.

### Inclusion and exclusion criteria

The inclusion criteria were: (1) peer-reviewed; (2) focused on FMDV vaccines; (3) targeted various animal species; (4) only articles in English; and (5) published between 2020 and 2025.

The exclusion criteria were: (1) studies addressing non-FMDV-related vaccines; (2) review articles; and (3) full text unavailable. Duplicate records identified during the search process were also removed.

### Data extraction

The data extraction process involved two levels of screening:

## Title and abstract screening

This preliminary step was conducted using Rayyan^35^ tool to screen the titles and abstracts of the articles to determine their relevance based on the predefined inclusion and exclusion criteria. Articles deemed irrelevant or duplicates were excluded at this stage **(**Fig. 1**)**.

## Full-Text screening and content evaluation

Articles that passed the first screening were subjected to a thorough evaluation of their full text. Two researchers read and analyzed the content to ensure alignment with the study’s objectives and criteria. Any disagreements were resolved through discussion, ensuring consistency in the selection process.

The form included fields for study details (author, year, and location), animal species, vaccine type, methodology, outcomes (e.g., immunogenicity and efficacy), and key conclusions. This ensured uniformity in data collection and allowed for accurate comparisons across studies.

### Statistical analysis

A meta-analysis was conducted using R version 4.3.0, utilizing the “meta”, “netmeta”, and “metafor” libraries. The objective was to estimate the pooled risk ratio (RR) with a 95% confidence interval (CI) for dichotomous outcomes (protected vs. non-protected animals). To address the heterogeneity of vaccine platforms, a random-effects model was employed to account for between-study variability, ensuring comparability across diverse designs and delivery methods^36^.

Due to the limited number of animals and the occurrence of zero-event studies, a continuity correction of 0.01 was applied to studies with zero events in one arm and subtracted from studies where all cases experienced protection. This smaller correction was chosen over the standard 0.5 to minimize bias in effect size estimation, particularly in small-sample FMD vaccine studies, where a larger correction could inflate risk ratios^34,37^.

Heterogeneity across studies was evaluated using the I² statistic (which describes the percentage of variation due to heterogeneity), tau-squared (τ²), and Cochran’s Q test (Chi-squared). Forest plots were generated to visualize individual study results and pooled estimates. Sensitivity analysis, including leave-one-out and exclusion of zero-event trials, addresses potential inflation of pooled effects due to small sample sizes and the continuity correction of 0.01 for zero-event studies. Leave-one-out sensitivity analysis was performed, removing each study sequentially to evaluate the influence of individual studies on pooled RR estimates. While zero-event sensitivity analysis excluded studies with zero events in one arm, recalculating the pooled RR and heterogeneity metrics to assess the robustness of the results^38^.

Subgroup analyses were defined by vaccine platform (peptide-based, VLP, viral vector, dendritic cell-based), with peptide-based vaccines including both single-epitope and dendrimeric peptide formulations grouped due to their shared reliance on synthetic peptide antigens, ensuring consistency in immunogenicity assessment across studies.

### Bibliometric analysis

To complement this comprehensive study, a bibliometric analysis was conducted to examine global research trends in FMDV vaccine development. Bibliographic records were retrieved from the Web of Science Core Collection, covering the period from 2000 to early 2025. The search was conducted in May 2025 and included all available peer-reviewed articles using the following query:

TS = (“foot-and-mouth disease virus” OR “FMDV”) AND TS = (“vaccine”).

For the bibliometric analysis, both original research articles and review papers were included, as the aim was to capture the breadth of scholarly output and collaboration patterns in the field. Conference abstracts, editorials, and book chapters were excluded. In contrast, for the systematic review and meta-analysis, only original research articles that met the eligibility criteria were included, with all reviews excluded.

Records were exported in BibTeX format and analysed using VOSviewer v1.6.18^39^ for keyword co-occurrence and collaboration networks, and Bibliometrix (R v3.1.4)^40^ for citation trends and productivity metrics.

This integration provides a comprehensive assessment of vaccine efficacy, research progress, and key knowledge gaps in the field of FMDV vaccine development.

## Results

**Fig. 1:**
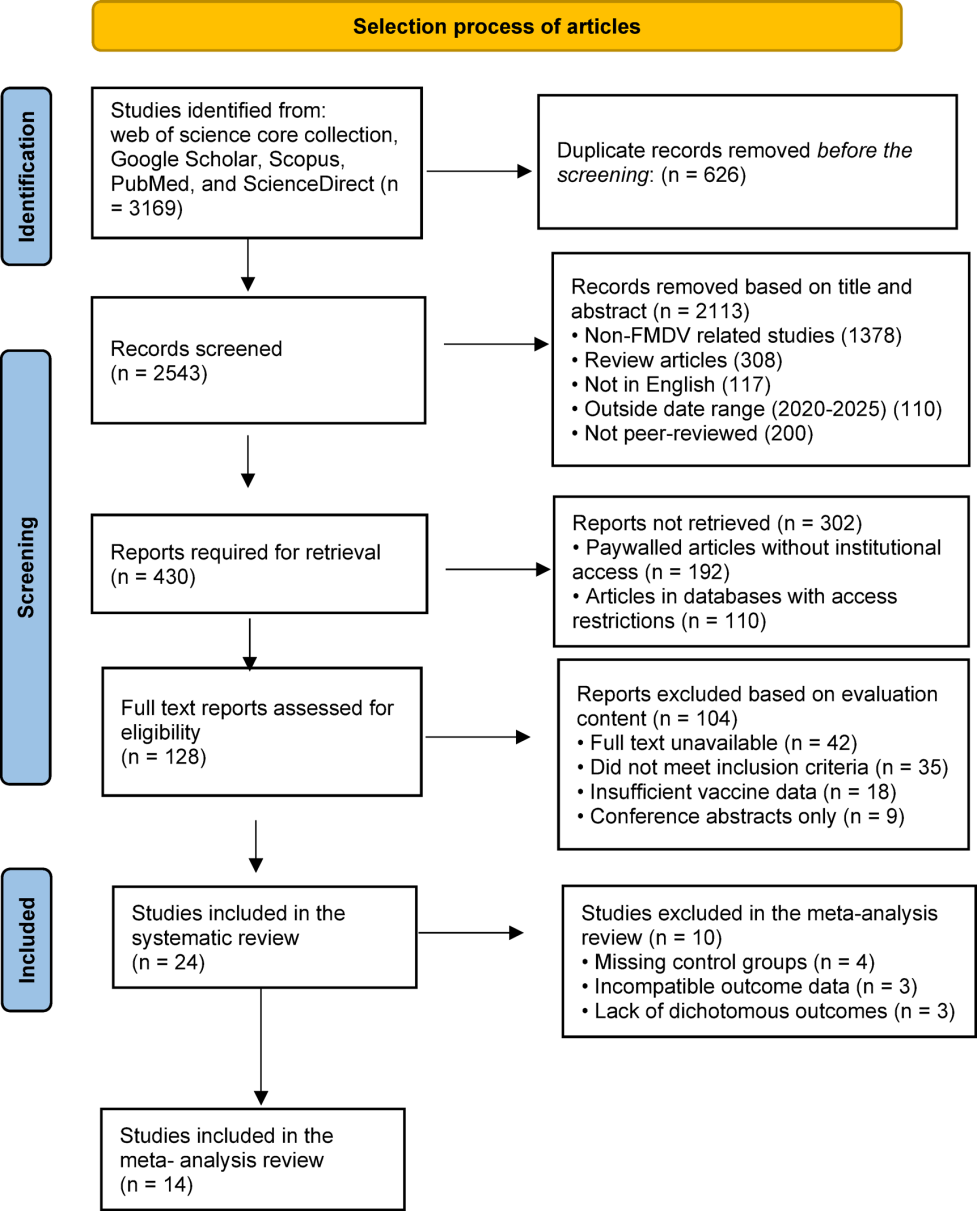
PRISMA flow diagram of search results.

A total of 3169 records were identified through database searching, including Web of Science, PubMed, Scopus, ScienceDirect, and Google Scholar. After removing 626 duplicates, 2543 records remained for title and abstract screening. Of these, 2113 were excluded for not meeting the inclusion criteria. The full texts of 128 articles were assessed for eligibility, resulting in 24 studies that met all inclusion criteria and were included in the qualitative synthesis (systematic review). Among these, 14 studies reported suitable, comparable dichotomous outcomes (number of protected vs. non-protected animals) and were therefore included in the quantitative meta-analysis. The remaining 10 studies were excluded from the meta-analysis due to missing control groups, lack of outcome data required for risk ratio calculations, or incompatible data formats. These studies, however, contributed relevant insights to the narrative review **(**Fig. 1**)**.

### Systematic review findings

The review evaluated twenty-four studies assessing the efficacy of FMD vaccines across various animal models, including guinea pigs, mice, pigs, and cattle. These studies were categorized by vaccine type to better understand the effectiveness of different vaccination strategies and their underlying mechanisms.

#### Peptide-Based vaccines

Multi-epitope vaccines are designed by combining multiple immunogenic fragments, typically B-cell epitopes that stimulate antibody production and T-cell epitopes that drive cellular responses, into a single recombinant construct. To maintain structural stability and proper antigen presentation, epitopes are often linked with short, flexible spacers, such as GGGGS or GEDG. Some designs also include universal T-cell helper sequences like PADRE or Invasin to broaden immunogenicity across different host species^26,41^. While these vaccines are considered safer and more scalable than traditional whole-virus formulations, their subunit nature typically requires the use of adjuvants to enhance immune responses. Reported adjuvant use varies, with some studies employing oil-based emulsions (e.g., Montanide) and others integrating adjuvant motifs directly into the recombinant construct.

Song et al. developed the 2BT-pIgG-Fc recombinant vaccine, containing two B-cell epitopes and one T-cell epitope from VP1 (135–178 aa) and 3D (341–370 aa). In guinea pigs, this vaccine conferred complete protection, and in pigs, it achieved a protection index (PI) of 90.43%, suggesting enhanced efficacy compared with commercial vaccines. The formulation was emulsified with Montanide ISA 201 adjuvant in a 50:50 (w/w) ratio (200 µg/dose intramuscular in guinea pigs, 1 mg/dose in pigs, two-dose schedules at 14- or 28-day intervals, respectively)^42^. The inclusion of the non-structural 3D epitope enhanced both humoral and cellular immune responses, highlighting the importance of combining structural and non-structural protein components.

Akter et al. evaluated two recombinant proteins (B1 and B3) incorporating G-H loop and C-terminal fragments from VP1 of serotypes O and A, plus PADRE and Invasin helpers linked with GEDG/C spacers. Immunization in guinea pigs induced dose-dependent protection, reaching 100% efficacy at 100 µg. The proteins were administered with Montanide ISA 201 adjuvant (1:1 w/w ratio) at doses ranging from 2 to 100 µg, highlighting the importance of both antigen design and adjuvant support^43^. This study emphasized the critical relationship between antigen dose and protective efficacy, providing valuable insights for vaccine optimization.

Moreover, a recent study developed a recombinant trivalent multi-epitope vaccine targeting three distinct topotypes of FMDV. Administered in pigs using a primary-booster schedule, the vaccine induced strong serotype O-specific neutralizing antibody responses and provided complete protection against all three topotypes. Notably, no anti-3ABC antibodies were detected in protected pigs, suggesting the induction of sterilizing immunity. The vaccine also maintained protective antibody levels for at least six months post-immunization, highlighting its potential as a long-lasting and broad-spectrum alternative for FMD control^44^.

Another novel bivalent recombinant multi-epitope vaccine targeting FMDV serotypes A and O was developed by fusing the HAO immunogen with heparin-binding hemagglutinin (HBHA), a TLR4 agonist acting as an intrinsic adjuvant, resulting in the HAO-HBHA fusion protein. This recombinant construct significantly activated dendritic cells (DCs) through the TLR4 pathway in both in vitro and in vivo mouse models. Immunization with HAO-HBHA elicited strong humoral responses (as confirmed by ELISA and virus neutralization tests) and enhanced cellular immunity, indicated by elevated cytokine production in Th1 and Th2 cells. These results observed that TLR4 agonist–based recombinant vaccines may offer a safe and effective strategy for multi-epitope-based protection against FMDV^45^.

Chathuranga et al. demonstrated the effectiveness of recombinant VP1-based vaccines against FMDV serotypes O and A in mice, achieving significant protection through targeted epitope presentation^22^. These peptide-based approaches offer advantages in terms of safety profile and manufacturing scalability compared to whole-virus vaccines. Zaher et al. reported the in silico design of a heptavalent multi-epitope vaccine, targeting top FMDV isolates in Egypt. Molecular docking predicted strong binding to bovine TLR-4 and TLR-2, paving the way for experimental validation^41^.

Patricia de León et al. explored innovative dendrimeric peptide platforms that displayed multiple copies of FMDV epitopes on branched molecular scaffolds. These vaccines induced high neutralizing antibody titres and robust IFN-γ production in pigs, revealing enhanced immunogenicity through multivalent antigen presentation^46,47^. Cañas-Arranz et al. further validated the B₂T(mal) dendrimeric peptide vaccine, composed of two copies of a B-cell epitope VP1(140–158) chemically linked to a T-cell epitope 3 A(21–35) via maleimide bonds. In pigs, two doses of B₂T(mal) with Montanide ISA 50V2 adjuvant elicited high titres of FMDV-specific IgG and neutralizing antibodies, sustained for 4–5 months, along with IFN-γ-producing T-cell responses. Notably, a single dose of B₂T(mal) also conferred long-lasting protection (up to 136 days post-boost), indicating its potential for reducing the need for frequent re-vaccination. The study also explored combining the vaccine with RNA transcripts from FMDV non-coding regions, which are known to enhance immune responses. These findings support B₂T(mal) as a strong candidate for safe, long-lasting FMDV peptide-based vaccination in swine^48–50^. Building upon earlier B2T dendrimer designs, Defaus et al. developed a novel multiple antigen peptide (MAP)-based vaccine construct termed B₂T-TB₂, in which two B2T units were fused in a tail-to-tail homodimeric configuration. This dendrimeric fusion was designed to enhance immune presentation and stability. Administered at a single 2 mg dose in pigs, the vaccine elicited robust B-cell and T-cell responses, with immunogenicity sustained for over four months. Remarkably, the B₂T-TB₂ formulation was observed to induce higher immune responses than earlier monomeric B2T prototypes and required a lower dose, suggesting its potential as a versatile, modular peptide-based vaccine for FMDV control in swine, the natural host^51^.

#### Virus-Like particle (VLP) vaccines

Virus-like particle vaccines are designed to closely resemble the natural 146 S intact virion, which represents the most effective protective antigen found in traditional inactivated FMDV vaccines. This 146 S structure consists of 60 protomers, each containing the viral proteins VP1, VP2, VP3, and VP4, which together form a complete capsid that presents the virus’s key conformational epitopes, features critical for triggering strong neutralizing antibody responses. When this structure dissociates into smaller 12 S subunits, much of its protective potential is lost. VLPs attempt to mimic the complete 146 S form while lacking viral genetic material, making them a safer yet highly immunogenic alternative. VLP vaccines combine the advantages of subunit vaccines with enhanced immunogenicity through structural mimicry of native virions^52^.

Giselle Rangel et al. developed chimeric Rabbit Hemorrhagic Disease Virus (RHDV) VLPs displaying FMDV epitopes, specifically targeting the VP1 B-cell epitope (140–158) and the T-cell epitope (3 A 21–35). This vaccine successfully induced neutralizing antibodies in mice and provided partial protection in pigs against FMDV serotype O. Of six immunized pigs, one was fully protected (lesions only at the inoculation site), while the remaining five developed secondary vesicles, but with delayed onset (3–4 days later than controls), reduced lesion severity, and significantly lower clinical scores (mean 4.66 ± 0.42 vs. 11.50 ± 0.50 in controls; *p* = 0.0031). None of the immunized animals showed the severe disease course observed in controls, highlighting that chimeric VLPs significantly reduced disease severity despite not providing complete protection^53^.

The VLP platform enhanced antigen presentation and processing, leading to improved immune recognition compared to soluble peptides. Moreover, a study described baculovirus-based VLPs with FMDV epitopes presented using the ADDomer system and co-administered with CD154. Piglets exhibited significantly enhanced immune responses, both humoral and cellular^54^.

Additionally, Li et al. successfully produced FMDV VLPs in *Pichia pastoris* by co-expressing the P1 capsid precursor and a mutated 3 C protease enhanced with an N‑terminal helix–loop–helix motif for better processing. Administering a single 50 µg intramuscular dose to mice and pigs induced strong FMDV-specific IgG and neutralizing antibodies, robust IFN‑γ cellular responses, and conferred 80–86% protection in pigs post-challenge compared to 0% in PBS-immunized controls, demonstrating both immunogenicity and partial protection in a cost-efficient yeast-expression system^55^.

The partial protection observed in pigs suggests that while VLP vaccines show promise, optimization of epitope selection and particle design may be necessary to achieve complete protection. This finding highlights the need for continued research into VLP-based platforms for FMDV vaccination.

#### Vectored vaccines

Viral vectored vaccines utilize modified viruses to deliver FMDV antigens, providing advantages in terms of immune stimulation and antigen presentation.

##### Adenoviral vectors

Xie et al. evaluated a replication-deficient human adenovirus type 5 (Ad5)-based vaccine expressing the capsid protein precursor P1-2 A and a mutated viral 3 C protease from FMDV strain A/GDMM/CHA/2013. This vaccine induced significant humoral and cellular immune responses in mice (1 × 10^8^ viral particles intramuscularly) and achieved a remarkable 100% protection in guinea pigs (1 × 10^9^ viral particles intradermally)^56^. The success of this approach demonstrates the potential of adenoviral vectors to enhance both the magnitude and breadth of immune responses.

##### Vesicular stomatitis virus (VSV) vectors

Lee et al. developed recombinant Vesicular Stomatitis Virus (VSV) glycoprotein carrying FMDV epitopes (VSV GP-VP1), which elicited significantly higher antibody responses compared to GST-VP1 fusion proteins and commercial vaccines^57^. This vectored approach leveraged the natural immunogenicity of VSV to enhance FMDV-specific immune responses.

##### *Salmonella* vector vaccine

Zhi et al. used irradiated *Salmonella Typhimurium* (KST0666) as a vector to deliver the VP1 protein of FMDV type A/WH/CHA/09. Mice immunized with this construct showed strong humoral (IgG, IgA) and neutralizing antibody responses, along with enhanced cellular immunity involving Th1, Th2, Th17, and activated CD8 + T cells. Immune responses were evaluated using FMDV VLPs under Biosafety Level (BSL-1) conditions, confirming the platform’s safety and effectiveness^58^.

#### Novel delivery systems and adjuvant technologies

Several studies explored innovative delivery mechanisms to enhance vaccine efficacy and practical application.

##### Nanoliposome delivery

Heshmati et al. encapsulated synthetic FMDV peptides (VP1 141–161 and 198–211 from serotype O/2016) into nanoliposomes prepared with cholesterol (Chol), 1,2-dimyristoyl-sn-glycero-3-phospho (DMPG), and 1,2-dimyristoyl-sn-glycero-3-phosphocholine (DMPC) lipids (5:4:16 molar ratio). The particles (~ 130 nm, 67% encapsulation efficiency) provided slow antigen release (< 2% in 24 h; complete over 7 days). In guinea pigs, intramuscular immunization (100 µg/dose, three doses at two-week intervals) induced FMDV-specific IgG responses. Compared to traditional adjuvants, Freund’s adjuvant generated the strongest response, followed by nanoliposomes and Alum. The study suggested that nanoliposomes improve peptide stability and controlled release, but still require optimization for potency^59^.

Also, An et al. evaluated dendritic mesoporous silica nanoparticles (DMSNs) as a self-adjuvanted delivery platform for the peptide vaccine B₂T against FMDV. Two DMSN sizes (57 nm and 156 nm) offered sustained, controlled B₂T release (up to ~ 930 h) in vitro and significantly enhanced in vivo immunogenicity in mice, eliciting high IgG titres comparable to Montanide™ formulations, with the 57 nm carriers showing the strongest response. This study demonstrated that DMSNs can prolong antigen release and enhance humoral immunity without exogenous adjuvants^60^.

##### Oral vaccine delivery

Zhang et al. engineered *Lactococcus lactis* to express multi-epitope proteins (TB1 and TB1-Co1) targeting intestinal M cells. TB1 contained epitopes from VP1 (21–60, 140–160, 200–212) and 3 A (21–35), while TB1-Co1 incorporated a Co1 ligand to improve uptake. Using the nisin-controlled expression (NICE) system, oral gavage in mice (1 × 10⁹ CFU at days 1, 11, and 21) and guinea pigs (1 × 10¹⁰ CFU, with/without CpG-ODN) induced mucosal secretory IgA, systemic IgG/IgA, neutralizing antibodies, and cytokines (IFN-γ, IL-2, IL-5, IL-10). In guinea pigs, this strategy provided ~ 60% protection post-challenge^61^. The findings highlight the potential of oral delivery for mass vaccination in resource-limited settings.

##### Chimeric vaccine platforms

Li et al. constructed a chimeric inactivated vaccine (rHN/TURVP1), inserting the VP1 gene of strain O/TUR/5/2009 into the O/HN/CHA/93 backbone. Formulated in a water-in-oil-in-water emulsion (12 µg/dose), the vaccine was tested in pigs and cattle (*n* = 18 each, intramuscular with a 28-day booster). It induced cross-neutralizing antibodies (> 1.65 log₁₀) against Mya-98, PanAsia, Ind-2001, and Cathay topotypes, with r₁ values > 0.3, suggesting an improved antigenic match^62^. This approach addresses one of the major challenges in FMDV vaccination, the need for cross-protection against diverse viral variants.

Building on this, Hwang et al. developed chimeric multi-topotype constructs, including PA2-VP1 and JC-VP1. PA2-VP1 incorporated VP1 from the ME-SA topotype (PanAsia-2 lineage; O/PAK/44/2008), while JC-VP1 contained the VP1 gene from the SEA topotype (Mya-98 lineage; O/SKR/Jincheon/2014), both inserted into the O Manisa backbone. Inactivated and formulated with ISA 206, saponin, and aluminum hydroxide (15–20 µg/dose), the vaccines conferred complete protection in mice, pigs, and cattle against South East Asia (SEA), Middle East South Asia (ME-SA), and Cathay topotypes. Between the two, PA2-VP1 induced higher and earlier neutralizing antibody titers than JC-VP1, underscoring the potential of chimeric vaccine platforms to overcome FMDV antigenic diversity^9^.

##### Extracellular vesicle (EV)–Based antigen delivery

Menay et al. reported a pioneering in vitro study whereby dendritic cells pulsed with inactivated FMDV (O1 Campos, 10 µg/mL for 16 h) secreted extracellular vehicles (EVs) of ~ 155 nm that express viral proteins, classical EV markers (CD9, CD63, CD81), and immunoregulatory molecules (MHC-II, CD86). These EVs induced significant FMDV-specific B-cell proliferation (including marginal-zone and follicular B cells) and indirectly stimulated T-cell responses in splenocytes from vaccinated mice. The findings indicate EVs may serve as natural antigen-carriers for both B- and T-cell activation, offering an innovative and potentially more stable alternative to conventional antigen and peptide delivery methods^63^.

**Fig. 2:**
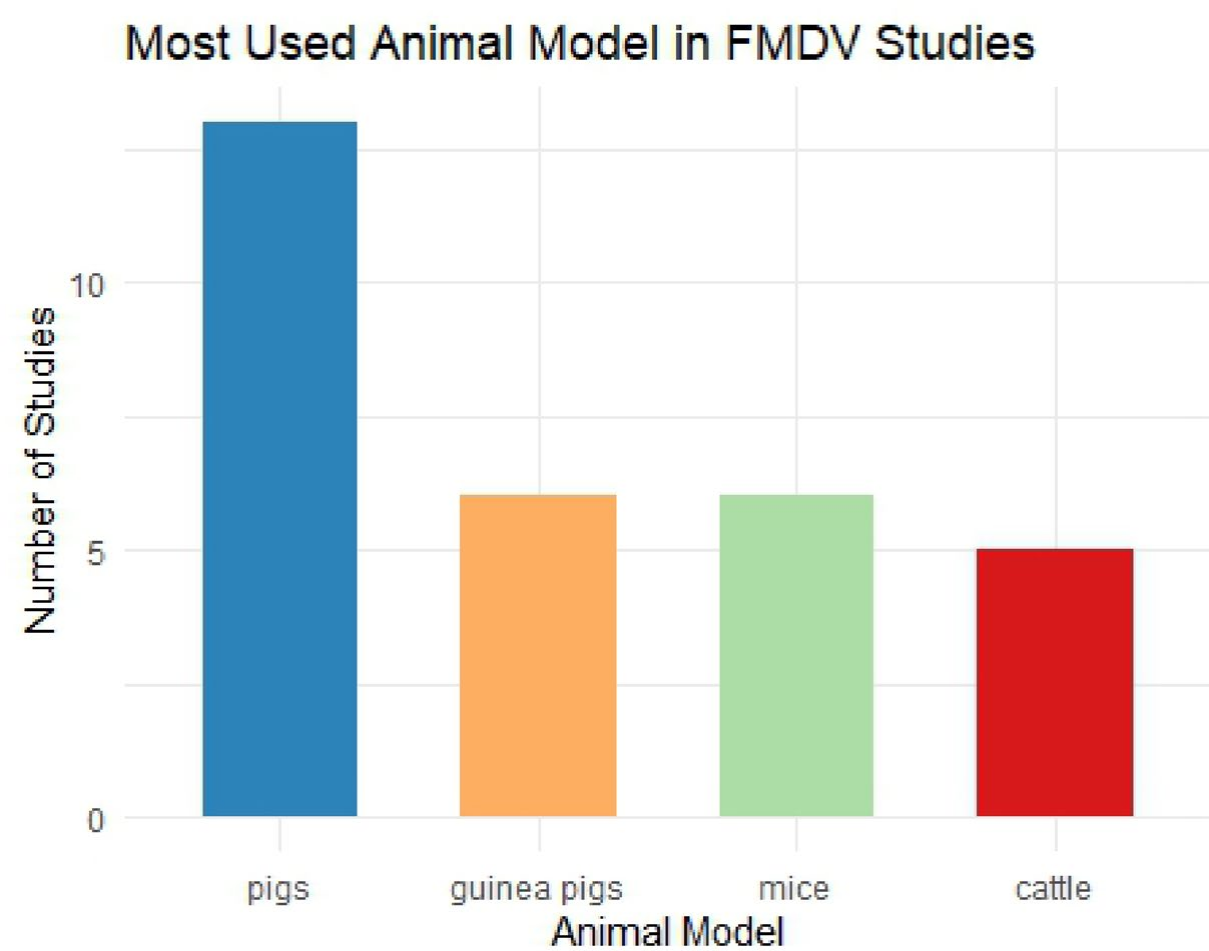
Illustration of the animal model most used in foot and mouth disease studies for vaccine evaluation. Pigs were the most frequently used model (13 studies, 54.2%), reflecting their high susceptibility to FMDV and relevance as natural hosts for vaccine efficacy assessment. Guinea pigs and mice were each used in 6 studies (25.0% each), serving primarily for preliminary immunogenicity screening and dose optimization studies. Cattle were utilized in 5 studies (20.8%), despite being a primary target species for FMDV vaccination in field conditions. The relatively limited use of cattle represents a significant research gap, as immune responses may differ between species, potentially affecting the translatability of vaccine efficacy results to target livestock populations. Some studies employed multiple animal models, contributing to multiple categories in this analysis.

This review underscores the progress in developing FMD vaccines, with a focus on antigenic proteins, serotype coverage, animal models **(**Fig. 2**)**, and novel vaccine delivery strategies^9,42,46,53,61^.

**Fig. 3:**
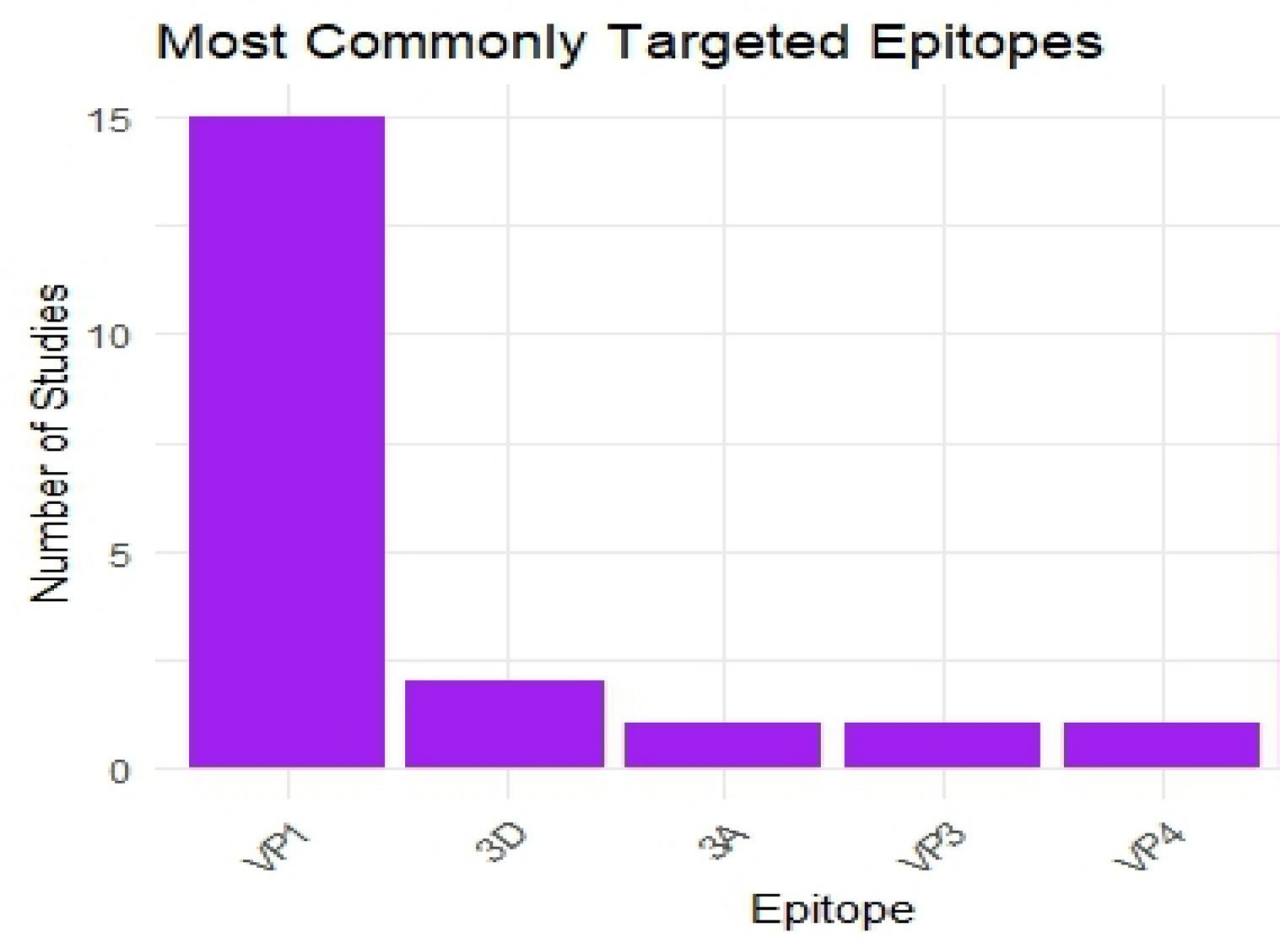
Bar chart that shows the most commonly targeted epitopes. VP1 epitopes were the predominant target, appearing in 15 studies (62.5%), reflecting their immunodominant properties and role in viral attachment. Non-structural protein 3D epitopes were utilized in 2 studies (8.3%), while structural proteins 3 A, VP3, and VP4 were each targeted in 1 study (4.2% each). The dominance of VP1 epitopes aligns with established knowledge of their critical role in neutralizing antibody induction, though the limited exploration of other epitopes suggests potential opportunities for broader antigenic coverage in future multi-epitope vaccine designs. Data represent unique epitope targets; studies utilizing multiple epitopes contributed to multiple categories.

While VP1 remains one of the most targeted proteins in FMDV vaccine research due to its immunodominant epitopes, particularly within the GH loop region (residues 130–160), it also presents several limitations **(**Fig. 3**)**. One major drawback is its high antigenic variability across serotypes and topotypes, which restricts the breadth of protection and limits its effectiveness in regions where multiple FMDV variants co-circulate^64,65^. As a result, VP1-based vaccines often exhibit serotype-specific immunity, requiring multivalent or multi-topotype formulations to achieve broader coverage. Additionally, some studies have shown that VP1 alone may not induce long-lasting immunity and that its immunogenicity can be influenced by conformational stability and presentation context (e.g., linear peptide vs. native-like structure). These challenges highlight the need to combine VP1 with conserved epitopes from other structural or non-structural proteins, or to use delivery systems (such as VLPs or dendrimers) that better preserve its native conformation.

### Meta-analysis findings

To complement the qualitative findings of the systematic review, a meta-analysis was conducted to quantitatively assess the protective efficacy of different vaccine platforms developed for FMDV between 2020 and 2025. The analysis included animal challenge studies reporting dichotomous outcomes (protection vs. no protection). Vaccine platforms analysed included peptide-based vaccines, VLP vaccines, viral vector vaccines, and DC-based vaccines **(**Table 1**)**.

**Table 1:** Characteristics and summary findings of the selected studies.

Study_ID	Vaccine_Type	Events_Tx	Total_Tx	Events_Ctrl	Total_Ctrl	Confidence Intervals (CI)
Rodrigo Cañas-Arranz 2020_single	Peptide based vaccine	8	12	0	2	**0.00–41,440,507,364
Rodrigo Cañas-Arranz 2020_booster	Peptide based vaccine	6	6	0	2	**0.00–61,805,363,664
Yinli Xie 2020	Viral vector vaccine	10	16	4	12	0.77–4.55
Yong Zhi 2021	Viral vector vaccine	5	5	0	5	**0.00–159,080,437,712
Zhiyao Li 2025_mice	VLP vaccine	5	5	5	10	1.07–3.71
Zhiyao Li 2025_pig	VLP vaccine	4	6	5	9	0.53–2.71
Giselle Rangel 2021_pig	VLP vaccine	1	6	0	2	**0.00–11,196,159,253
Giselle Rangel 2021_mice	VLP vaccine	25	25	0	5	**0.00–159,329,462,173
Salma Akter2024	Peptide based vaccine	23	25	6	9	0.86–2.22
W. A.Gayan Chathuranga 2022_mice	Peptide based vaccine	5	5	5	10	1.07–3.71
W. A.Gayan Chathuranga 2022_pig	Peptide based vaccine	7	8	0	6	**0.00–168,205,050,929
Sung Ho Shin 2024	Peptide based vaccine	12	12	0	6	**0.00–191,739,361,075
Byeong-Min Song 2024_guinea pigs	Peptide based vaccine	3	3	0	3	**0.00–94,088,297,976
Byeong-Min Song 2024_pigs	Peptide based vaccine	5	5	0	5	**0.00–159,080,437,712
Seong Yun Hwang 2023	Peptide based vaccine	22	24	0	9	**0.00–265,399,725,582
Pinghua Li 2022_pig	Peptide based vaccine	9	12	6	6	0.54–1.04
Pinghua Li 2022_cattle	Peptide based vaccine	9	12	6	6	0.54–1.04
Fudong Zhang 2021	Peptide based vaccine	9	15	0	5	**0.00–96,053,601,622
Suyu Mu 2024	DC-based vaccine	3	3	0	3	**0.00–94,088,297,976

** Wide confidence intervals in some studies reflect zero events in control groups, handled through standard statistical procedures for meta-analysis.

**Fig. 4:**
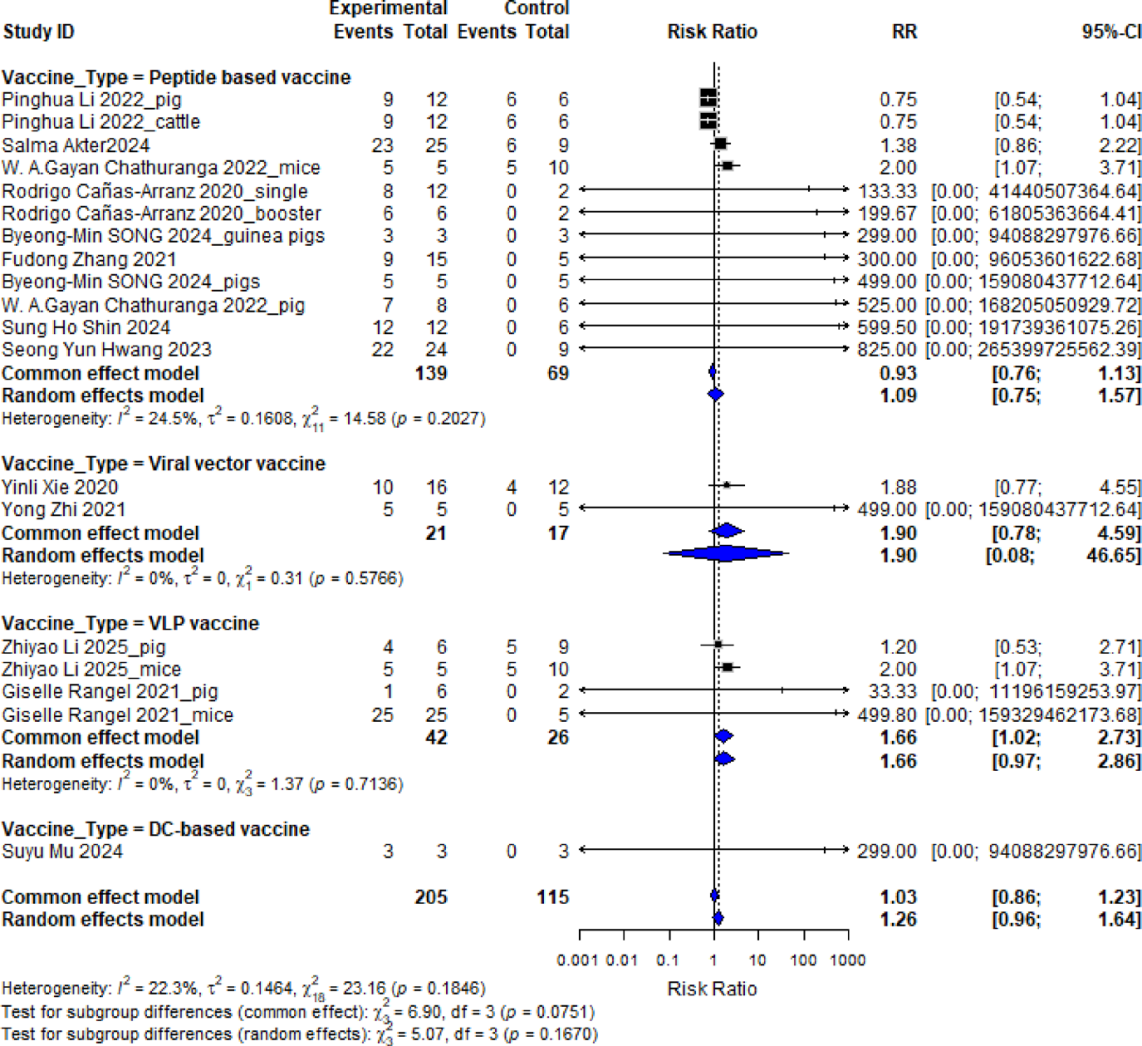
A subgroup Forest plot analysis displaying individual study risk ratios (RR) with 95% confidence intervals (CI) for protection outcomes (protected vs. non-protected animals) across vaccine types (peptide-based, VLP, viral vector, and dendritic cell-based). Each square represents a study estimate, sized by weight; horizontal lines show CIs. Subgroup means are diamonds. RR > 1 indicates higher protection in vaccinated vs. control groups. Heterogeneity is quantified by I² (percentage of variation due to heterogeneity) and tau-squared (τ²).

Across all included studies, the total number of experimental animals was 205, compared to 115 control animals. The RR compares the likelihood of protection between the vaccinated and control groups. A value greater than 1 suggests the vaccine improves protection, a value of 1 indicates no difference, and a value less than 1 suggests a disadvantage. The CI gives a range where the true effect likely falls; if the interval includes 1.0, the result is not statistically significant. The pooled estimate for all vaccine types yielded a RR of 1.26 with a 95% CI of 0.96 to 1.64, indicating a slight but statistically non-significant increase in protection among vaccinated animals. To better understand performance across platforms, subgroup analysis was conducted **(**Fig. 4**)**.

To allow for variability across studies, a random-effects model was used. This model assumes that the effect size may differ between studies due to differences in design, population, or vaccine formulation. To assess consistency across studies, heterogeneity was measured using three key statistics. First, the I² statistic indicates the percentage of variability due to real differences rather than chance; values under 25% are considered low, suggesting reliable consistency. Second, τ² estimates the variance across true effects in the population. Lastly, the Chi-squared (χ²) test assesses whether the observed variation exceeds what would be expected randomly.

The peptide-based vaccine group showed a pooled RR of 1.09 (95% CI: 0.75–1.57). This reflects a small overall protective effect compared to controls. The heterogeneity across the peptide vaccine studies was low, as indicated by an I² value of 24.5%, suggesting that most variation in effect sizes was due to real differences between studies rather than random chance. The between-study variance (τ²) was 0.1608, and the Chi-squared test for heterogeneity was non-significant (χ² = 14.58, degrees of freedom = 11, *p* = 0.2027), indicating an acceptable level of consistency in the findings **(**Fig. 4**)**.

Other vaccine platforms showed varied degrees of protection. VLP vaccines demonstrated a pooled RR of 1.66 (95% CI: 0.97–2.86) with subgroup heterogeneity statistics of I² = 0%, τ² = 0, χ² = 1.37, and *p* = 0.7136. Viral vector vaccines showed a higher pooled RR of 1.90 (95% CI: 0.08–46.65) with I² = 0%, τ² = 0, χ² = 0.31, and *p* = 0.0576, though the wide confidence interval reflects the limited number of studies available for this platform. Dendritic cell-based vaccines were represented by a single study, resulting in limited statistical power and wide confidence intervals.

**Fig. 5:**
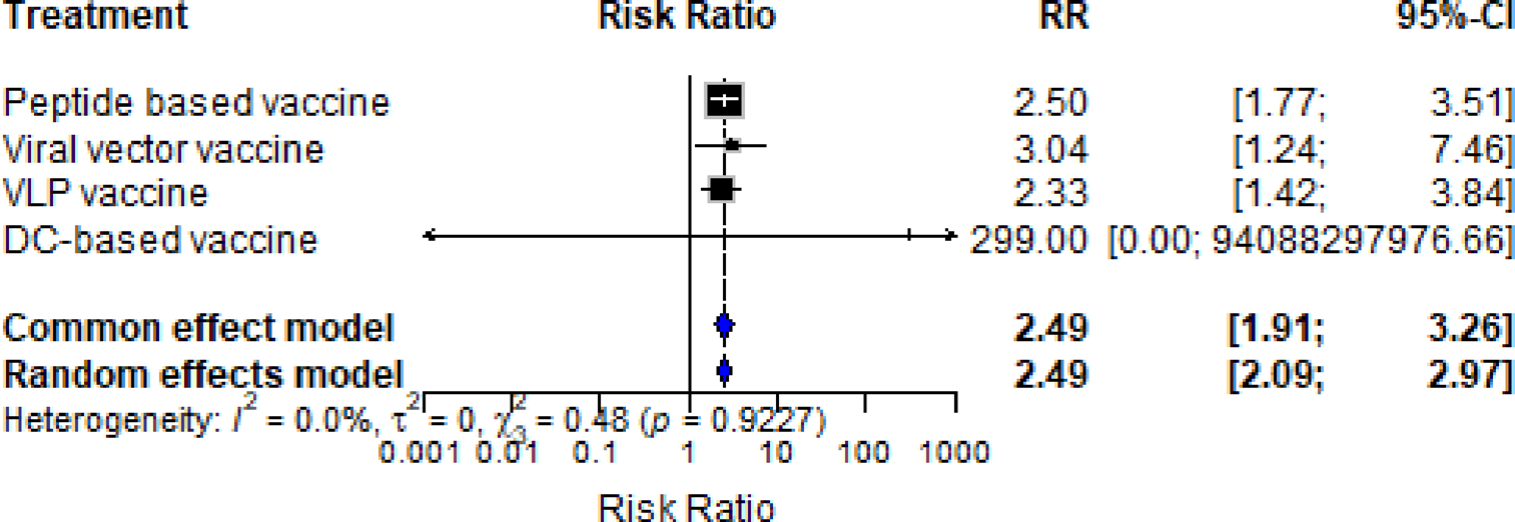
Forest plot displaying the overall pooled risk ratios (RR) with 95% confidence intervals (CI) for each vaccine platform, using a random-effects model to account for between-study variability. Each square represents a study estimate, sized by weight; horizontal lines show CIs. Pooled estimates are diamonds. RR > 1 favors the vaccine group over controls for protection outcomes. Heterogeneity is assessed via I² and tau-squared (τ²). The plot summarizes protective efficacy across platforms regardless of between-study variation.

The combined-treatment model displays the overall pooled RR for each vaccine platform regardless of between-study variation **(**Fig. 5**)**. The viral vector vaccine had the highest combined RR at 3.04, followed by peptide-based vaccines at 2.50, and VLP vaccines at 2.33. These results may appear more favourable, but they are subject to inflation from studies with zero-event control groups or limited sample sizes, and they do not reflect study-level heterogeneity.

Sensitivity analysis excluding zero-event control studies revealed mixed effects on estimate precision. Peptide-based vaccines showed RR = 1.06 (95% CI: 0.50–2.22), viral vector vaccines RR = 1.88 (95% CI: 0.77–4.55), and VLP vaccines RR = 1.65 (95% CI: 0.07–37.42). No analysis was possible for DC-based vaccines due to the removal of the single study. While point estimates remained relatively stable for all platforms, confidence intervals varied considerably, with viral vector vaccines showing improved precision and VLP vaccines displaying increased uncertainty (**Figure ****S1**). Leave-one-out sensitivity analysis confirmed these patterns **(Figures ****S2****−13)**.

### Bibliometric analysis findings

To contextualize the progress of FMDV vaccine development, a bibliometric analysis was conducted on 620 studies to identify global research trends, key contributors, and emerging themes.

#### Average citation per year

**Fig. 6:**
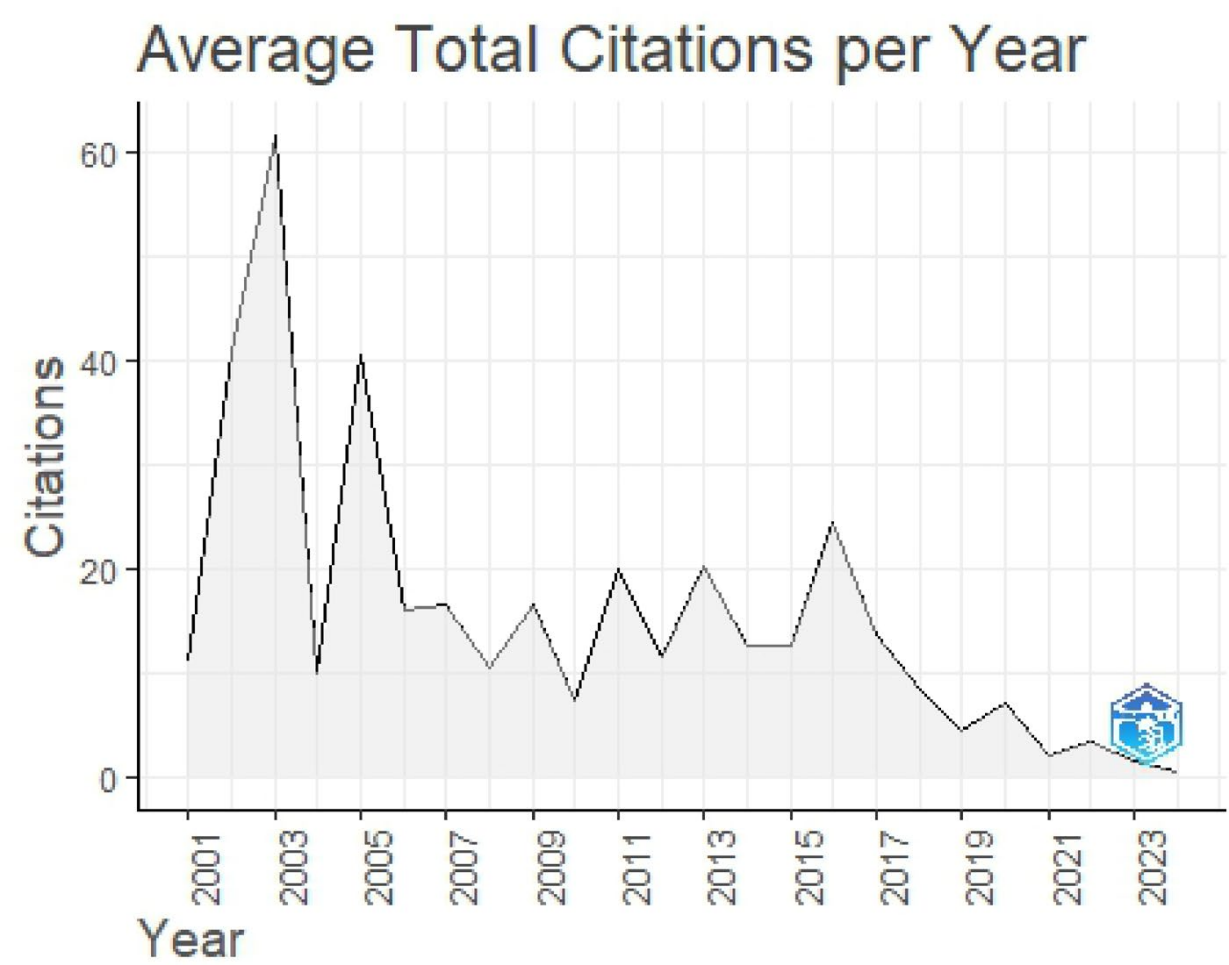
Fluctuation chart that illustrates the average total citations per year.

The average total citations per year for research related to FMDV highlights trends in the field’s academic impact over time **(**Fig. 6**)**. Citations saw a sharp rise in the early 2000 s, reaching a peak around 2003, suggesting a rise in research activity or the publication of highly influential studies during that period. However, after this peak, citation numbers began to decline, though with some fluctuations. From 2010 onward, the citation rate remained relatively stable but at a lower level compared to the peak years. In more recent years (2020–2023), citations have reached their lowest point, possibly indicating a shift in research priorities, fewer groundbreaking publications, or a natural decline in citations as older studies become less frequently referenced. This trend reflects how scientific focus and influence evolve within the field of FMDV research.

#### Global distribution of the FMDV research activation

**Fig. 7:**
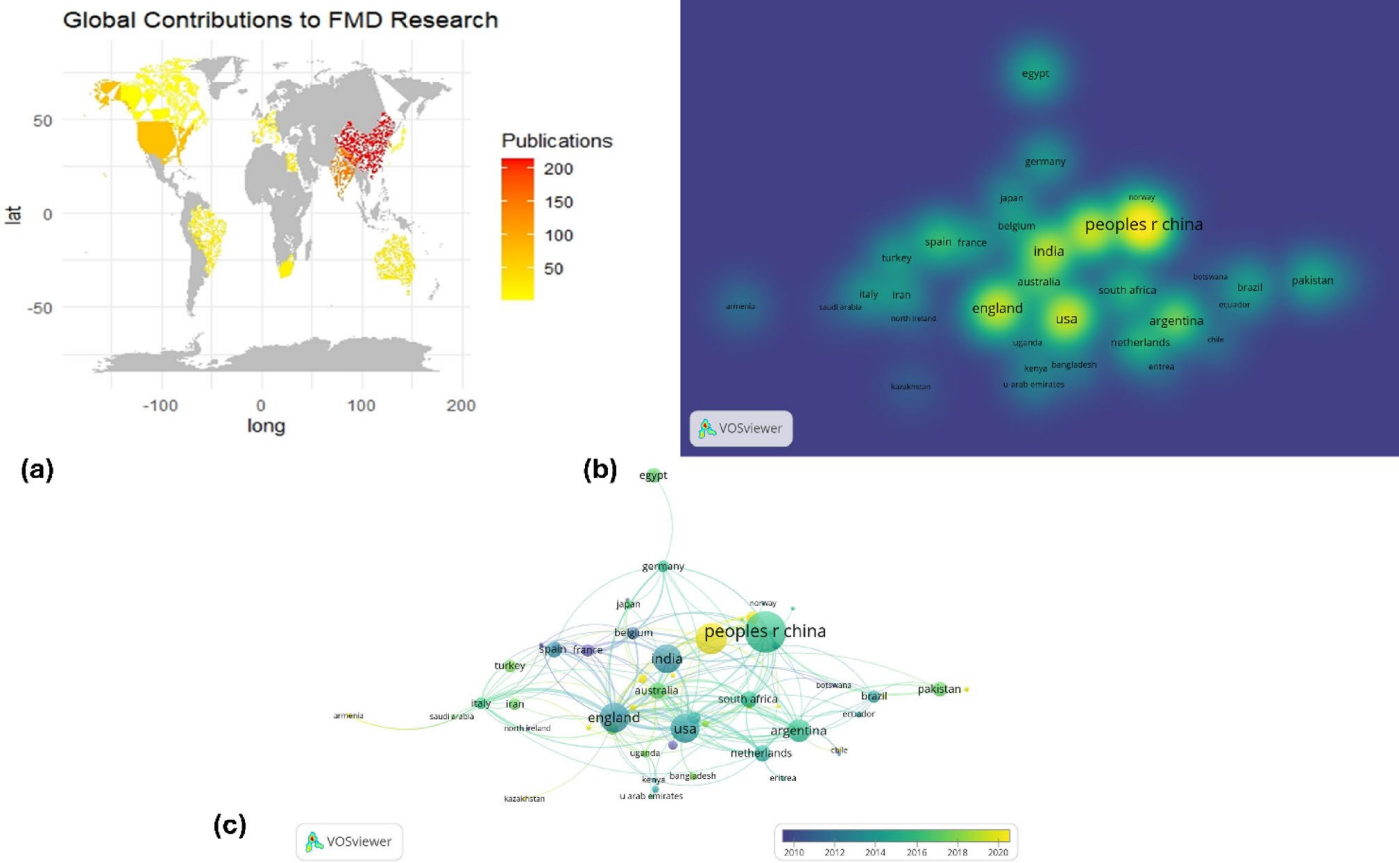
Heatmap illustration of the global distribution of the FMDV research (a), the intensity of research collaborations and publication volume by country (b), and the country collaboration network using VOSviewer (c).

The global heatmap of FMDV research contributions illustrates the number of publications from different regions **(**Fig. 7a**)**. Countries shaded in yellow to red represent higher research output, with China, the United States, and the United Kingdom being the most active contributors. This distribution reflects the global interest in FMDV research, particularly in regions with significant livestock industries where the disease poses an economic threat. Additionally, a density visualization from VOSviewer represents the intensity of research collaborations and publication volume by country **(**Fig. 7b**)**. The size and brightness of a country’s name correspond to its research impact. China, India, the USA, and England appear as the most influential contributors, with strong research networks extending across Europe, South Asia, and Africa. The presence of multiple countries across different continents indicates the collaborative nature of FMDV research, which is crucial for developing effective control strategies and vaccines.

#### Country collaboration network

The country collaboration network was generated using VOSviewer. It visualizes global research collaborations, showing that China, India, the USA, and England are central hubs with strong international connections. Countries like Egypt, Germany, and Pakistan have fewer links, indicating their independent or less-integrated research efforts. The color-coded clusters represent different regional collaborations, reflecting how various countries contribute to global FMD research **(**Fig. 7c**)**.

## Keyword co-occurrence network

**Fig. 8:**
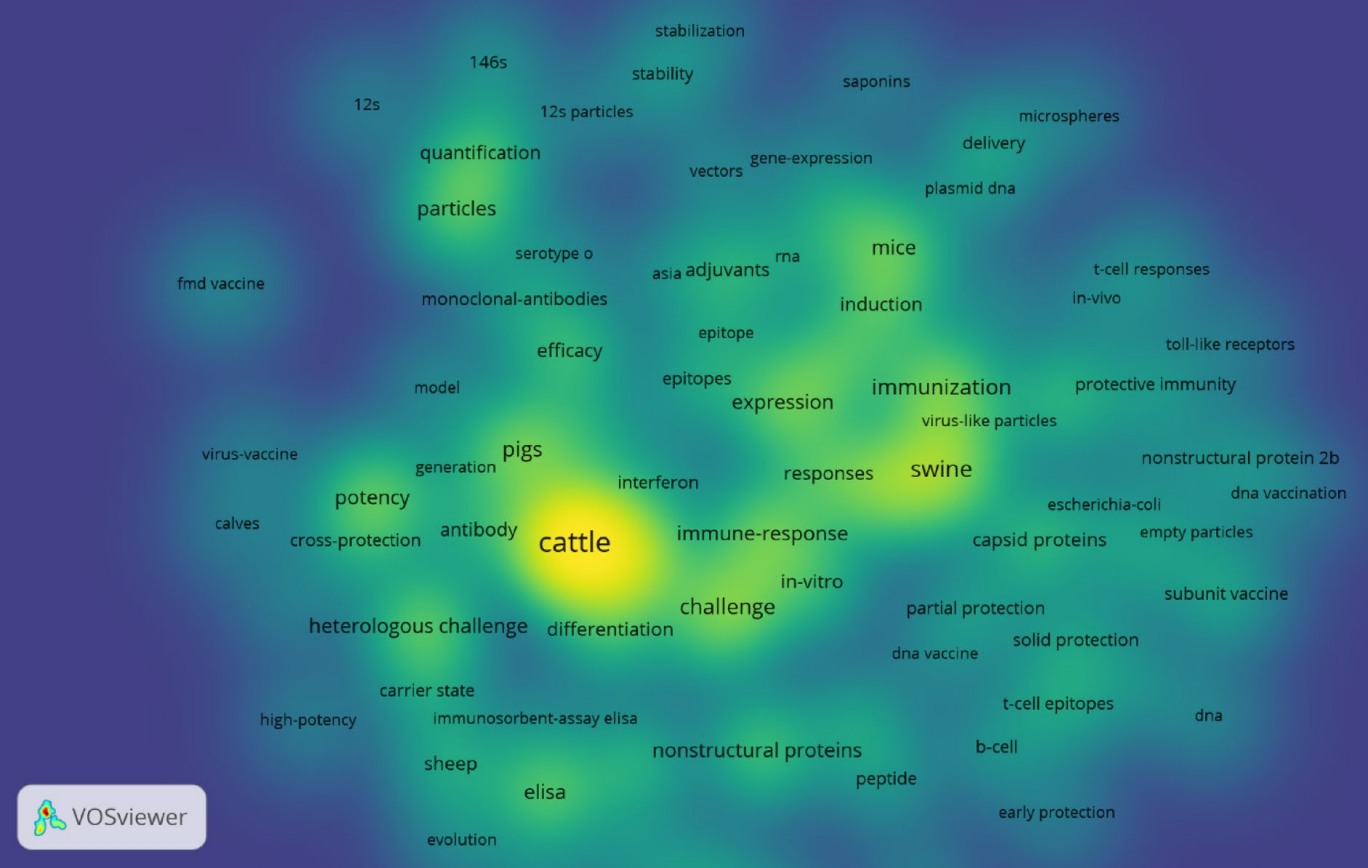
Keyword co-occurrence intensity plot illustrating research hotspots from 2020–2025 using VOSviewer.

Keyword co-occurrence analysis reveals research hotspots that can be directly correlated with protective efficacy outcomes from our meta-analysis. The bibliometric visualization shows “virus-like particles,” “capsid proteins,” and “146s” as prominent high-density clusters (bright green-yellow regions), indicating intensive research focus. This bibliometric prominence aligns with VLP vaccines achieving the second-highest pooled efficacy in our meta-analysis (RR = 1.66, 95% CI: 0.97–2.86) and demonstrating consistent results across studies (I² = 0%). Similarly, “viral vector,” “gene-expression,” and “plasmid DNA” appear as significant clusters in the keyword network, corresponding to viral vector vaccines showing the highest pooled risk ratio (RR = 1.90, 95% CI: 0.08–46.65) in our efficacy analysis. Peptide-related terms (“epitopes,” “peptide,” “nonstructural proteins”) occupy moderate-density regions in the bibliometric landscape, which corresponds to their moderate but stable protective efficacy (RR = 1.09, 95% CI: 0.90–1.33) with acceptable heterogeneity in our meta-analysis **(**Fig. 8**)**.

Interestingly, the country collaboration network observed in our analysis bears a strong resemblance to the World Organisation for Animal Health (WOAH) FMDV reference laboratory network. This alignment suggests that global research partnerships often mirror existing surveillance and diagnostic frameworks, with leading contributors such as China, the United Kingdom, and the United States playing central roles in both research output and laboratory capacity. Such overlap underscores the practical relevance of bibliometric patterns in understanding how global FMD control efforts are coordinated.

## Discussion

### Strengths

This review highlights the evolving landscape of FMD vaccine development, emphasizing the diversity of platforms, delivery strategies, and animal models evaluated in recent years. Peptide-based vaccines demonstrated consistent immunogenicity across species but often required higher doses or multiple administrations to achieve protective efficacy. The incorporation of NSP epitopes, particularly 3D, significantly enhanced both antibody production and T-cell responses, partially addressing the limitations of conventional subunit formulations^8^.

VLP vaccines, which structurally mimic the native virus, consistently showed stronger immunogenicity than soluble subunit vaccines. However, levels of protection varied between studies, highlighting the need for optimization in epitope design, particle size, and structural stability^52^. Vector-based vaccines, particularly those using viral or bacterial vectors to deliver VP1, elicited robust immune responses, often with a single dose, making them promising candidates for rapid immunization, especially during outbreaks^56^.

Across platforms, VP1 remained the central antigenic target^22,62^, with studies also exploring VP2, VP3, and VP4, as well as conserved NSPs to enhance breadth and durability^9,42^. Most vaccines focused on serotypes O and A, which are globally prevalent and economically significant, with several chimeric or multi-topotype designs achieving strong cross-protection in pigs and mice^66^.

The inclusion of diverse animal models provided a broad understanding of vaccine performance. Pigs emerged as the most representative and reliable model, while guinea pigs and mice were useful for preliminary assessments and dose optimization^42,43,46^. Innovative delivery systems, including nanoliposomes, dendrimeric peptides, and recombinant *Lactococcus lactis*, further enhanced antigen presentation and immune responses. The use of adjuvants such as Montanide ISA 50V2 and oil emulsions improved antigen stability and immunogenicity, supporting their role in vaccine optimization^59,61,67^.

The meta-analysis provided a quantitative complement to the systematic review, allowing comparison of different FMDV vaccine platforms. All three platforms, peptide-based, VLP, and viral vector vaccines, showed risk ratios above 1.0 compared to controls, indicating protective potential. Peptide-based vaccines demonstrated relatively low heterogeneity, especially in studies using larger doses or boosters, though their effect sizes were smaller overall. VLP vaccines showed consistent estimates with moderate protection, while viral vector vaccines yielded the highest point estimates but with wide confidence intervals, reflecting the limited number of available studies. Taken together, these findings suggest that while peptide vaccines remain promising, VLP and viral vector platforms may offer greater protective efficacy, although confirmation with larger datasets is needed.

Finally, the keyword co-occurrence analysis highlights research hotspots that align closely with the protective outcomes observed in our synthesis. Terms such as “virus-like particles,” “capsid proteins,” and “146S” formed high-density clusters, reflecting the strong global focus on VLP-based strategies and their consistent performance across experimental studies. Similarly, the prominence of “viral vector,” “gene expression,” and “plasmid DNA” in the bibliometric landscape corresponds to growing evidence of vector platforms as promising candidates for FMD vaccination. Peptide-related terms, including “epitopes” and “nonstructural proteins,” appeared in moderate-density regions, mirroring their role as an actively explored yet still evolving approach. In contrast, dendritic cell–based vaccines received minimal bibliometric emphasis, which parallels the more limited efficacy evidence available. Together, these patterns suggest that research attention, as captured by bibliometrics, broadly reflects vaccine platforms with emerging or demonstrated protective potential, while also highlighting areas where experimental validation lags behind scientific interest.

However, critical gaps emerge when examining research-efficacy relationships. While “cattle” appears prominently in bibliometric networks, only 20.8% of efficacy studies included cattle, representing a significant translation gap between research interest and practical validation. This disconnect is particularly concerning given cattle’s central role in FMD control programs.

### Limitations

The limited number of eligible studies in this review reflects several factors specific to FMDV vaccine research. First, FMDV research requires high-containment biosafety facilities, restricting the number of institutions able to conduct such studies. Second, our focus on the 2020–2025 period was intentional, as it captures the most recent advances in emerging vaccine platforms; this is particularly important given FMDV’s mutagenic nature, which necessitates up-to-date vaccine assessments. Third, our strict inclusion criteria ensured that only studies with sufficient experimental data, including challenge studies, and with robust methodology and appropriate controls were included; many publications were excluded because they lacked sufficient data for meaningful comparison^24,25,68^. Finally, FMDV vaccine studies are often species-specific, resulting in smaller, specialized study pools. To mitigate this limitation, we complemented the systematic review and meta-analysis with a bibliometric study, which provides a broader context on global research activity and trends in FMDV vaccine development.

Most vaccine studies focused on pigs or guinea pigs, with fewer trials in cattle, the primary target species. This reduces generalizability, as species-specific immune differences may limit extrapolation to the field. Publication bias toward positive results and the restriction to English-language studies may also have influenced outcomes. Moreover, meta-analysis findings for some platforms, particularly viral vectors, were based on small sample sizes, leading to wide confidence intervals and greater statistical uncertainty.

Another limitation is the predominance of serotypes O and A in the literature, with far less attention given to SAT1–SAT3 and Asia1, which remain relevant in endemic regions. This restricts the comprehensiveness of current vaccine strategies.

Additionally, the logistical challenges of vaccine delivery in resource-limited regions remain a significant barrier to widespread adoption. One of the most critical barriers to the widespread deployment of FMDV vaccines is the dependence on cold-chain infrastructure, which increases costs and limits accessibility in rural or low-income settings. Traditional inactivated vaccines, and even some recombinant formulations, require refrigeration to maintain antigen stability^8^. This poses challenges for consistent vaccine delivery, especially in regions with unreliable electricity or transportation systems. To address this, thermostable formulations are being actively explored. For instance, lyophilized (freeze-dried) peptide or VLP-based vaccines can maintain potency at ambient temperatures, allowing storage and transport without refrigeration. Additionally, dry powder intranasal or oral formulations, often combined with mucosal adjuvants like chitosan, are under investigation to reduce the need for trained personnel and sterile injection tools^69–73^.

### Future directions

Future FMDV vaccine research should prioritize multivalent and multi-topotype designs that provide cross-protection across diverse serotypes, particularly the understudied SAT and Asia1 lineages. Expanding trials in cattle is critical to validate findings from small animal models and ensure practical field application.

Advances in thermostable formulations using promising strategies include nanoparticle encapsulation, which not only protects antigens from degradation but also improves immune presentation. Technologies such as microneedle patches, although still in early stages for veterinary use, offer the potential for dose-sparing, pain-free delivery that does not require cold storage^74^. Expanding research and investment into these thermostable and field-friendly platforms will be essential to ensure that FMDV vaccines are both immunologically effective and practically deployable in remote endemic regions.

Bibliometric findings highlight that global research activity mirrors the platforms with promising experimental data (e.g., VLPs and viral vectors). Continued integration of bibliometrics with systematic and meta-analytic approaches can guide strategic investment into the most impactful vaccine platforms.

By strengthening cross-species validation, expanding to neglected serotypes, and adopting thermostable and scalable delivery systems, FMDV vaccines can advance toward broader protection and improved field deployment, supporting long-term disease control within the One Health framework.

## Conclusion

This systematic review and meta-analysis assessed recent developments in FMDV vaccine research between 2020 and 2025. The specialized nature of FMDV vaccine research, which requires high biosafety facilities and involves multiple animal species, naturally limits the number of available studies and contributes to methodological diversity across platforms. The meta-analysis revealed distinct patterns of statistical performance across vaccine platforms, with VLP and viral vector vaccines exhibiting risk ratios above 1.0, while peptide-based vaccines demonstrated more modest effects. Peptide-based vaccines, particularly those incorporating VP1 epitopes, demonstrated variable outcomes depending on formulation, dose, and delivery method, reflecting the complexity of optimizing these approaches. Dendritic cell-based vaccines were represented by insufficient data for meaningful assessment.

The bibliometric analysis revealed research trends that generally corresponded with meta-analysis findings, with high research intensity around VLP and viral vector approaches. However, critical research gaps were identified, particularly the underrepresentation of cattle studies despite their prominence in the bibliometric landscape, and limited focus on SAT1-SAT3 serotypes in recent literature. These findings highlight systematic gaps in FMDV vaccine evaluation that extend beyond individual study limitations to reflect broader research priorities and resource allocation patterns in the field.

The current evidence base, while limited by the specialized nature of FMDV research, provides valuable guidance for advancing vaccine development toward more effective global FMD control strategies. Future research priorities should focus on addressing the identified gaps through larger, standardized trials in cattle and expanded serotype coverage.

## Supplementary Information

Below is the link to the electronic supplementary material.

Supplementary Material 1

Supplementary Material 2

Supplementary Material 3

## Data Availability

All data generated or analysed during this study are included in this published article.

## Abbreviations

FMD: Foot and mouth disease
FMDV: Foot and mouth disease virus
VLP: virus-like particle
