# Supplementary material for: Systematic review and meta-analysis of the effectiveness of polypeptide, virus-like particles, and viral vector vaccines for foot-and-mouth disease (2020–2025)
Source: Sci Rep. 2025 Nov 10;15:39370. doi: 10.1038/s41598-025-24078-5 (PMC12603288; doi:10.1038/s41598-025-24078-5)
Supplement: Supplementary file 3 — Supplementary Material 3 [file 41598_2025_24078_MOESM3_ESM.docx]

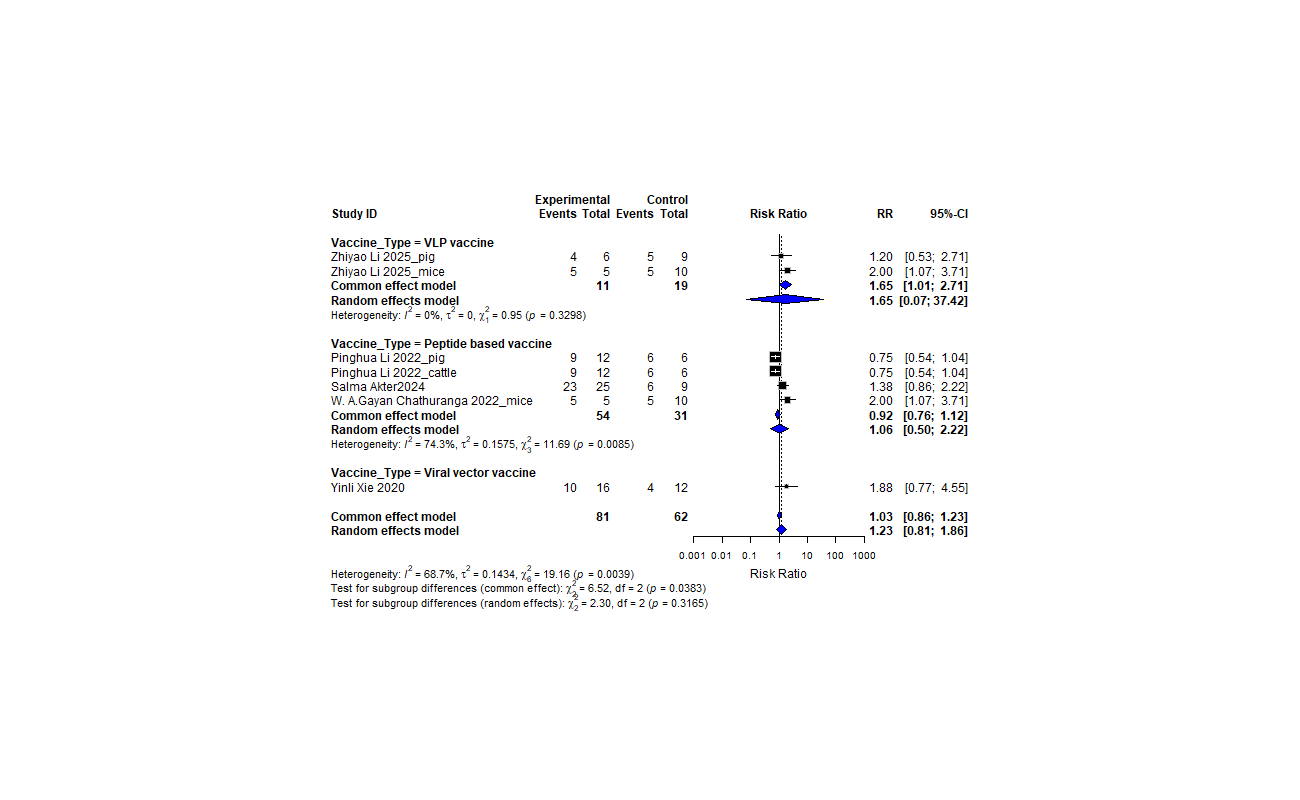


Fig. S1: Forest plot of sensitivity analysis excluding studies with zero events in the control arm (12 studies removed from 19 total), showing pooled risk ratios (RR) with 95% confidence intervals (CI) for protection outcomes across vaccine platforms using a random-effects model. No analysis is possible for DC-based vaccines due to the removal of the single study. Squares represent individual study RR, with sizes proportional to weight, and diamonds indicate pooled estimates.


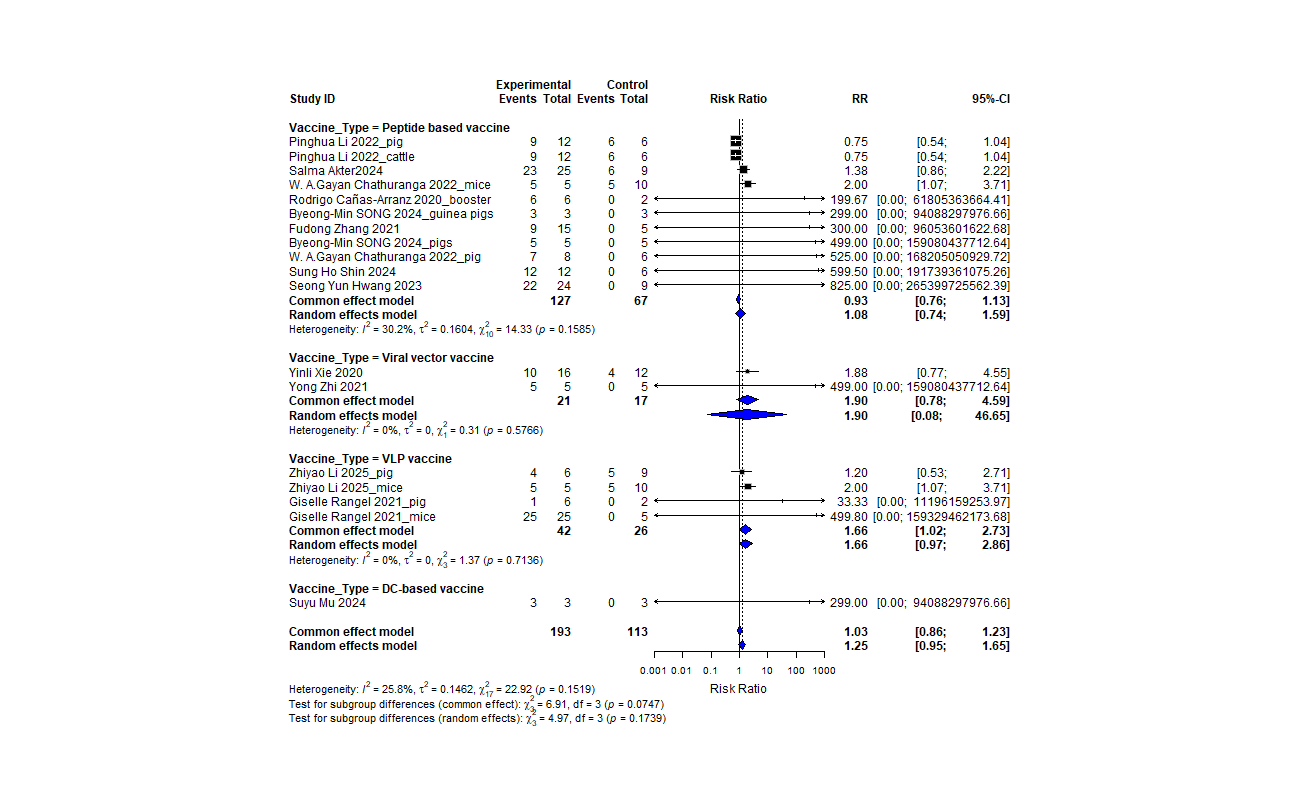


Fig. S2: Forest plot of leave-one-out sensitivity analysis removing one study at a time and recalculating the pooled effect size (Rodrigo Cañas-Arranz 2020_single study removed from 19 total), showing pooled risk ratios (RR) with 95% confidence intervals (CI) for protection outcomes across vaccine platforms using a random-effects model.


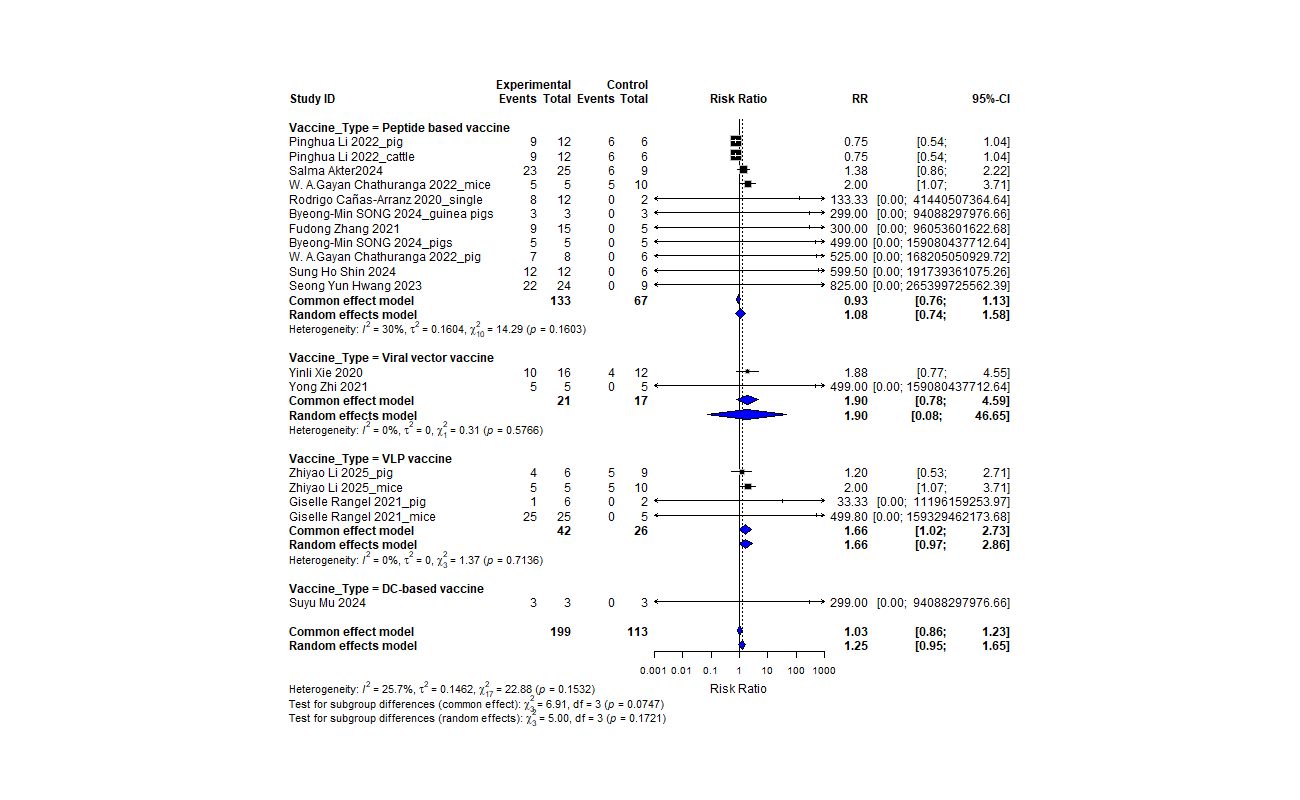


Fig. S3: Forest plot of leave-one-out sensitivity analysis removing one study at a time and recalculating the pooled effect size (Rodrigo Cañas-Arranz 2020_booster study removed from 19 total), showing pooled risk ratios (RR) with 95% confidence intervals (CI) for protection outcomes across vaccine platforms using a random-effects model.


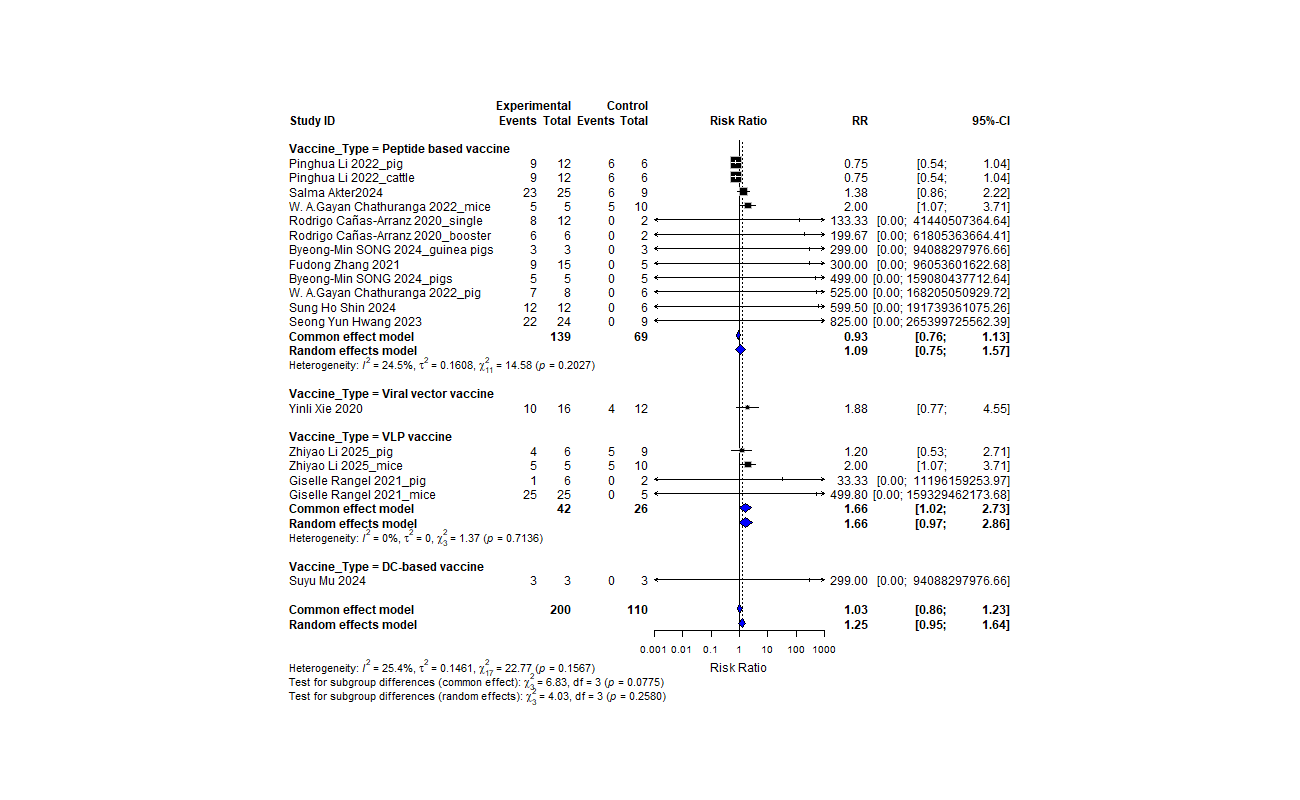


Fig. S4: Forest plot of leave-one-out sensitivity analysis removing one study at a time and recalculating the pooled effect size (Yong Zhi 2021 study removed from 19 total), showing pooled risk ratios (RR) with 95% confidence intervals (CI) for protection outcomes across vaccine platforms using a random-effects model.


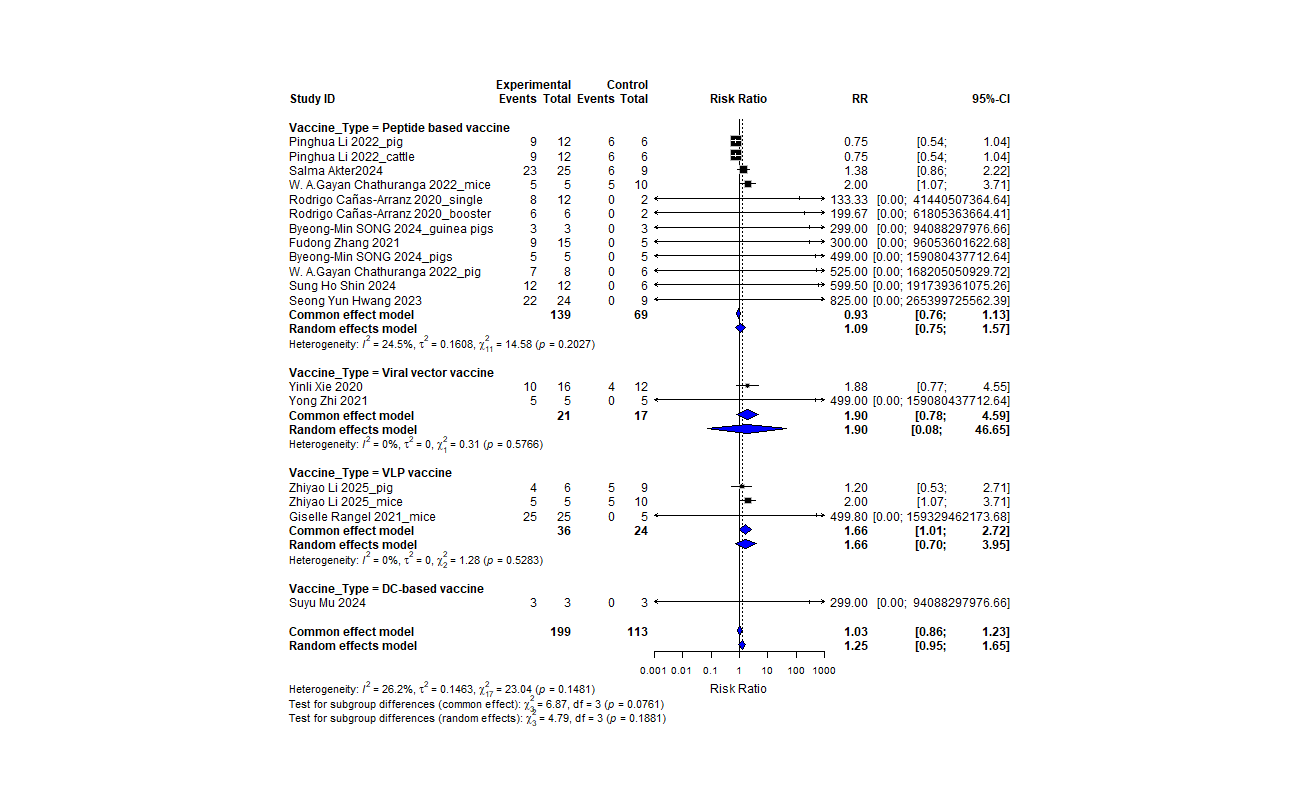


Fig. S5: Forest plot of leave-one-out sensitivity analysis removing one study at a time and recalculating the pooled effect size (Giselle Rangel 2021_pig study removed from 19 total), showing pooled risk ratios (RR) with 95% confidence intervals (CI) for protection outcomes across vaccine platforms using a random-effects model.


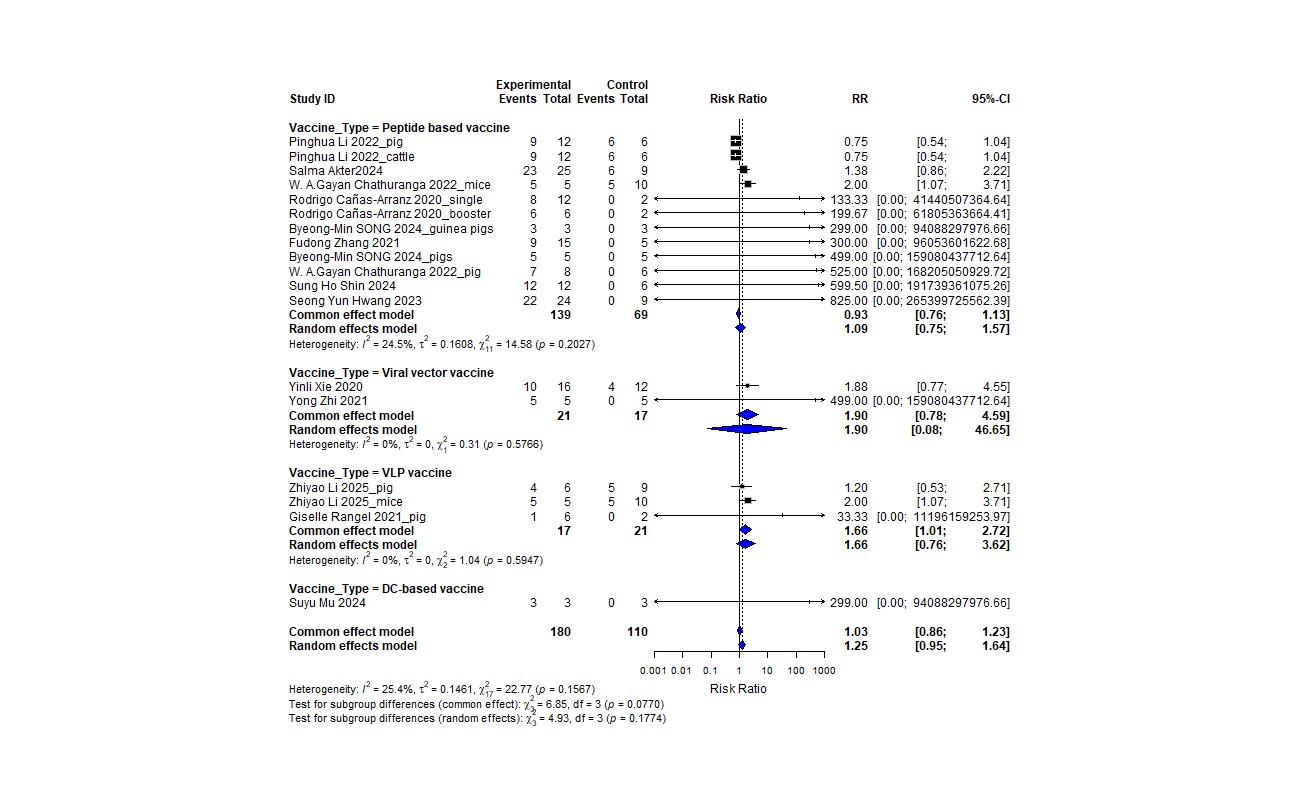


Fig. S6: Forest plot of leave-one-out sensitivity analysis removing one study at a time and recalculating the pooled effect size (Giselle Rangel 2021_mice study removed from 19 total), showing pooled risk ratios (RR) with 95% confidence intervals (CI) for protection outcomes across vaccine platforms using a random-effects model.


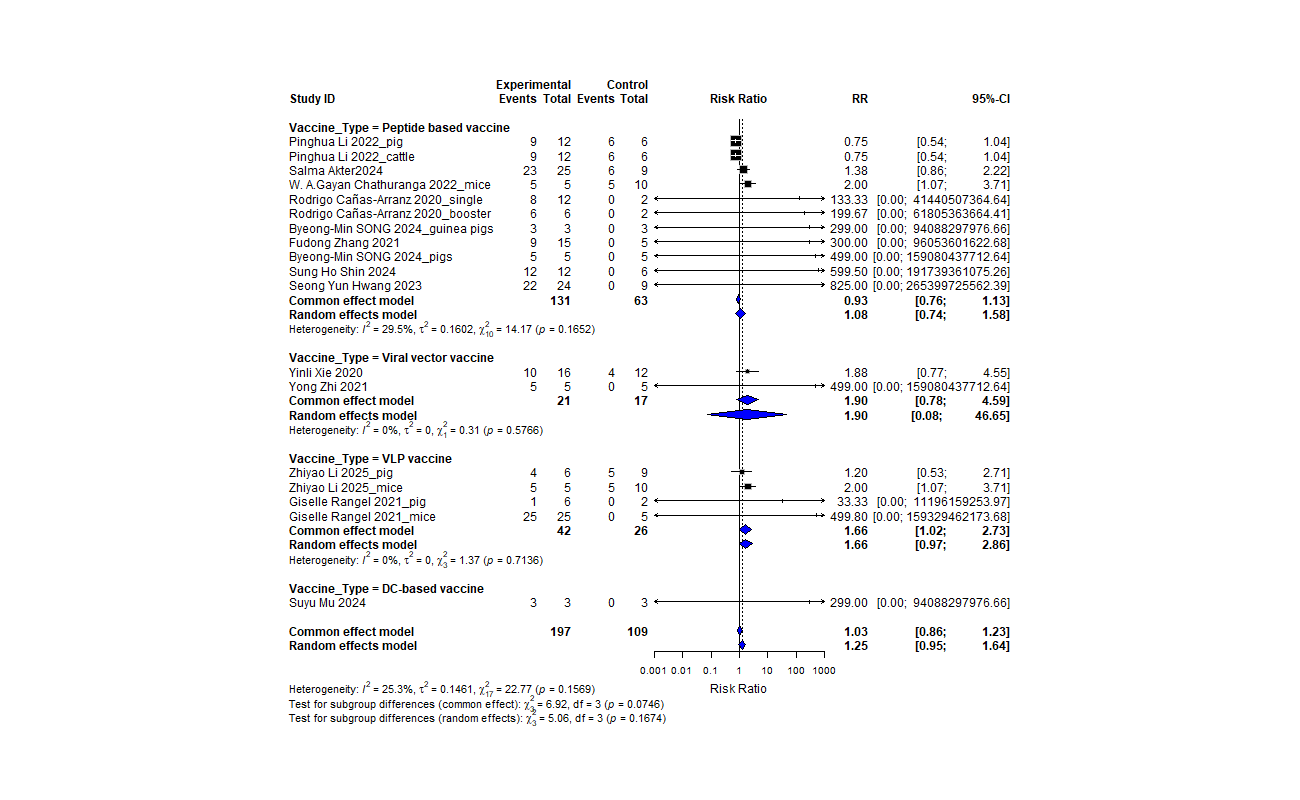


Fig. S7: Forest plot of leave-one-out sensitivity analysis removing one study at a time and recalculating the pooled effect size (W. A.Gayan Chathuranga 2022_pig study removed from 19 total), showing pooled risk ratios (RR) with 95% confidence intervals (CI) for protection outcomes across vaccine platforms using a random-effects model.


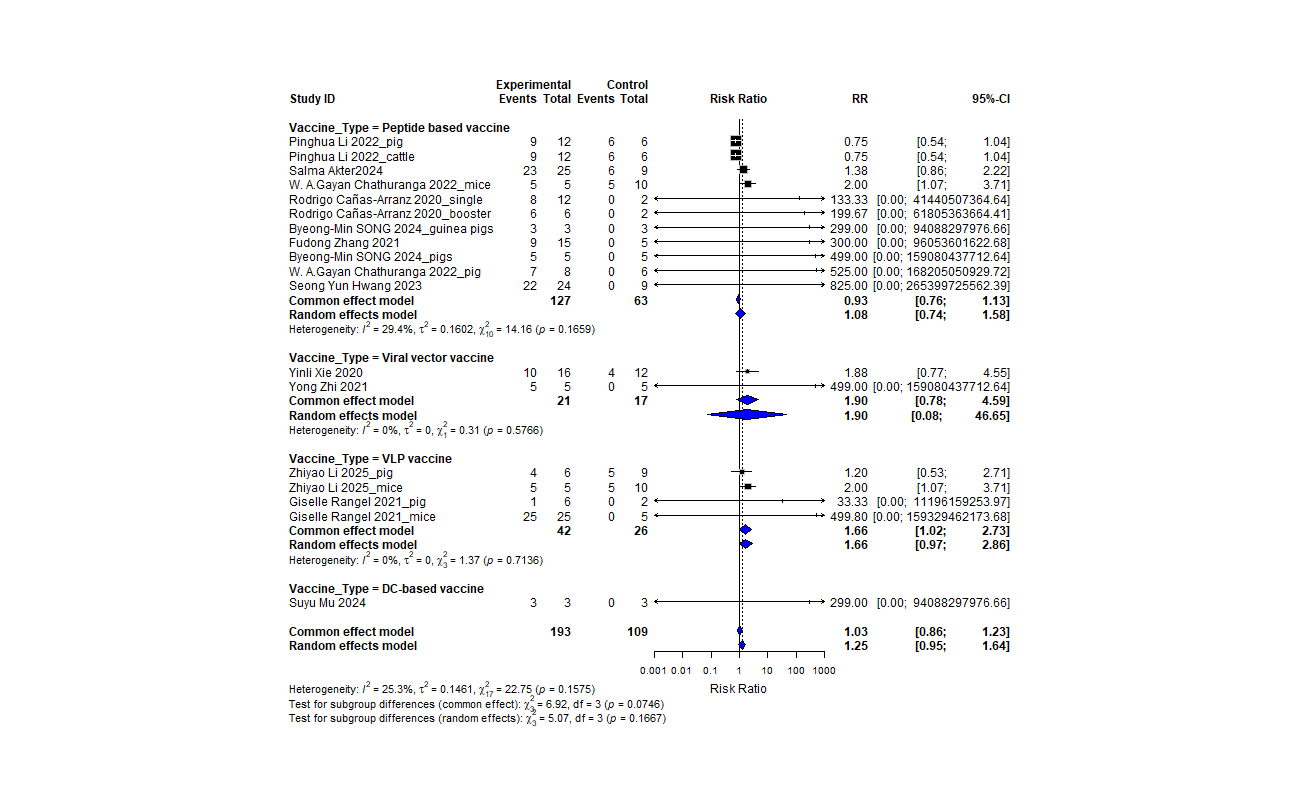


Fig. S8: Forest plot of leave-one-out sensitivity analysis removing one study at a time and recalculating the pooled effect size (Sung Ho Shin 2024 study removed from 19 total), showing pooled risk ratios (RR) with 95% confidence intervals (CI) for protection outcomes across vaccine platforms using a random-effects model.


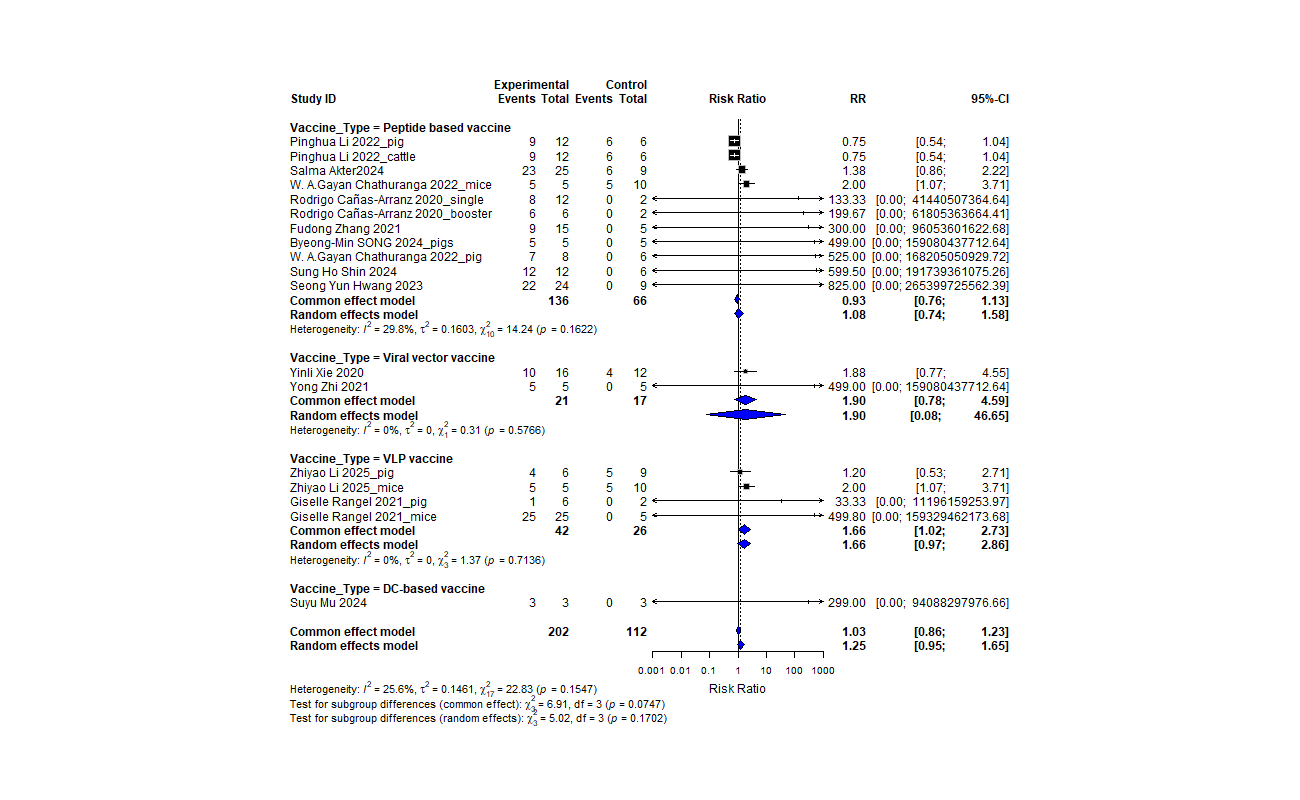


Fig. S9: Forest plot of leave-one-out sensitivity analysis removing one study at a time and recalculating the pooled effect size (Byeong-Min SONG 2024_guinea pigs study removed from 19 total), showing pooled risk ratios (RR) with 95% confidence intervals (CI) for protection outcomes across vaccine platforms using a random-effects model.


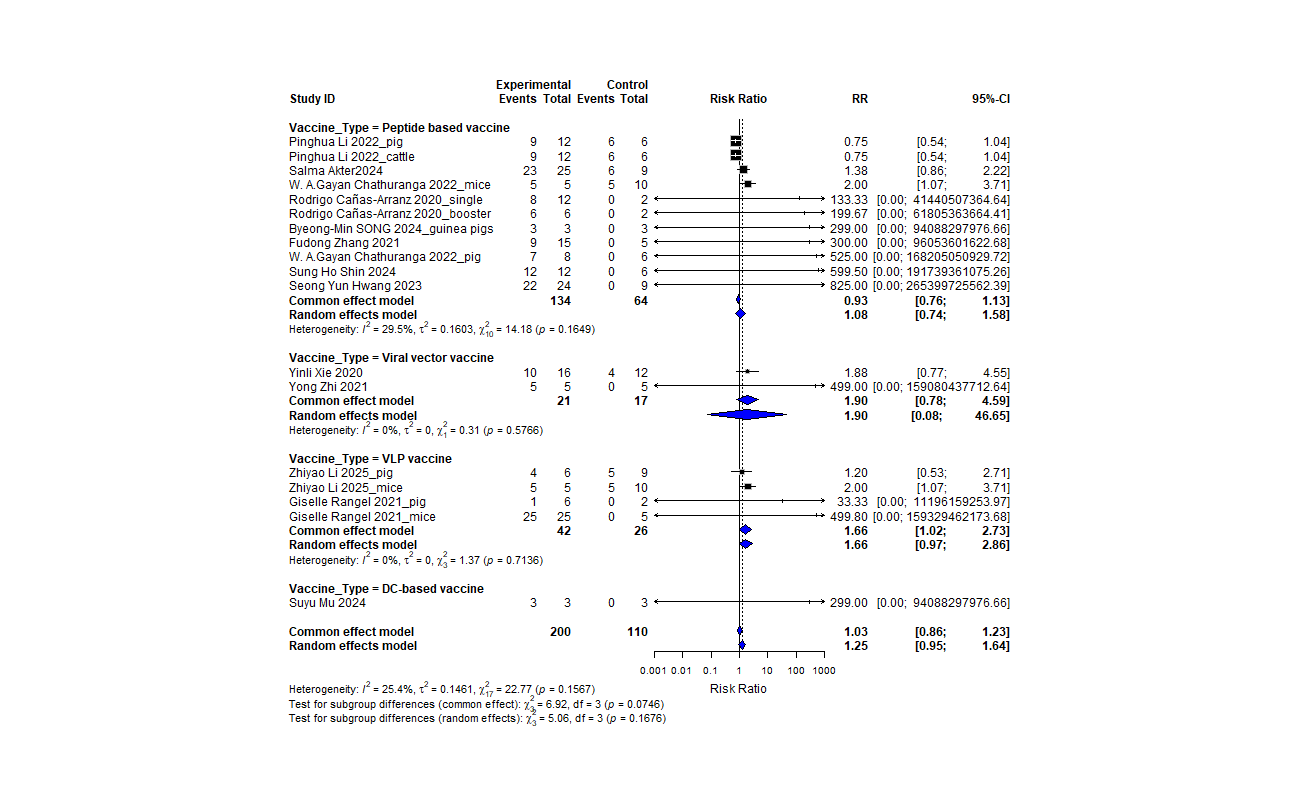


Fig. S10: Forest plot of leave-one-out sensitivity analysis removing one study at a time and recalculating the pooled effect size (Byeong-Min SONG 2024_pigs study removed from 19 total), showing pooled risk ratios (RR) with 95% confidence intervals (CI) for protection outcomes across vaccine platforms using a random-effects model.


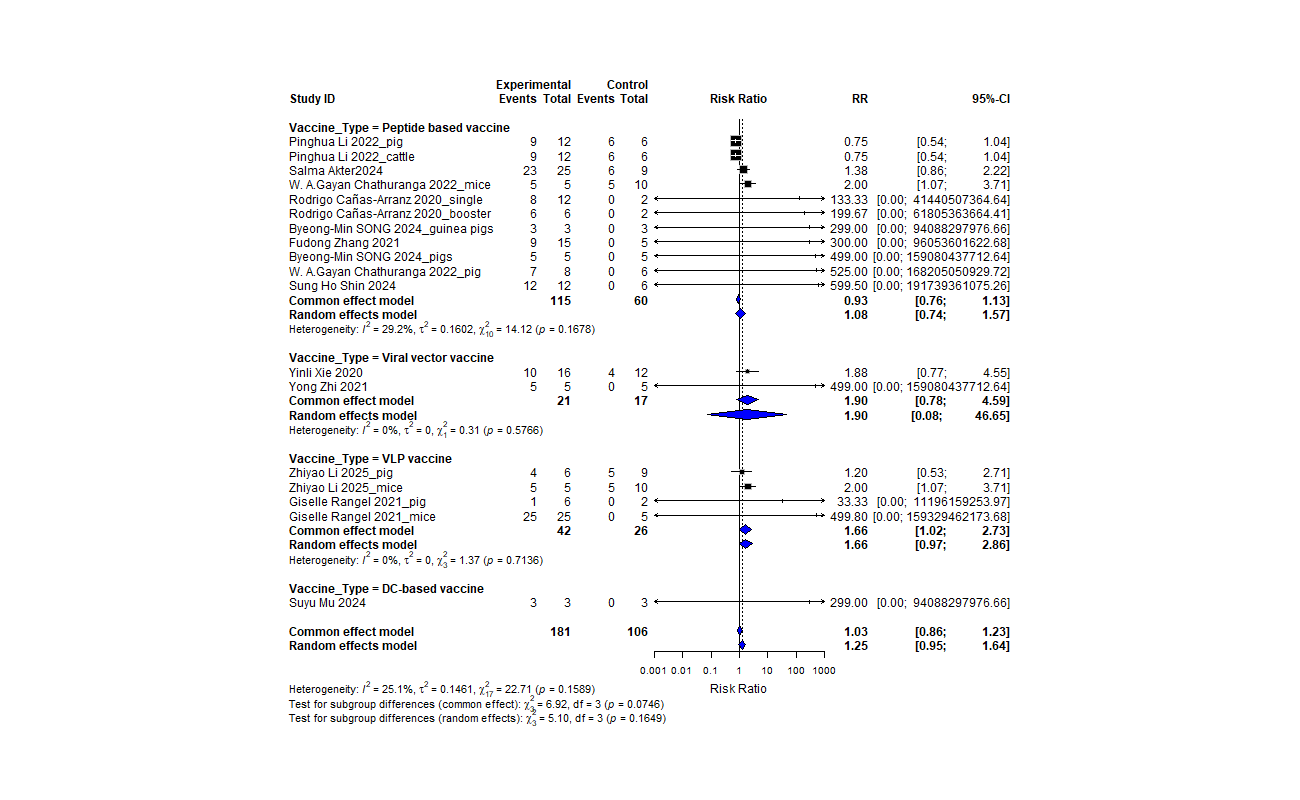


Fig. S11: Forest plot of leave-one-out sensitivity analysis removing one study at a time and recalculating the pooled effect size (Seong Yun Hwang 2023 study removed from 19 total), showing pooled risk ratios (RR) with 95% confidence intervals (CI) for protection outcomes across vaccine platforms using a random-effects model.


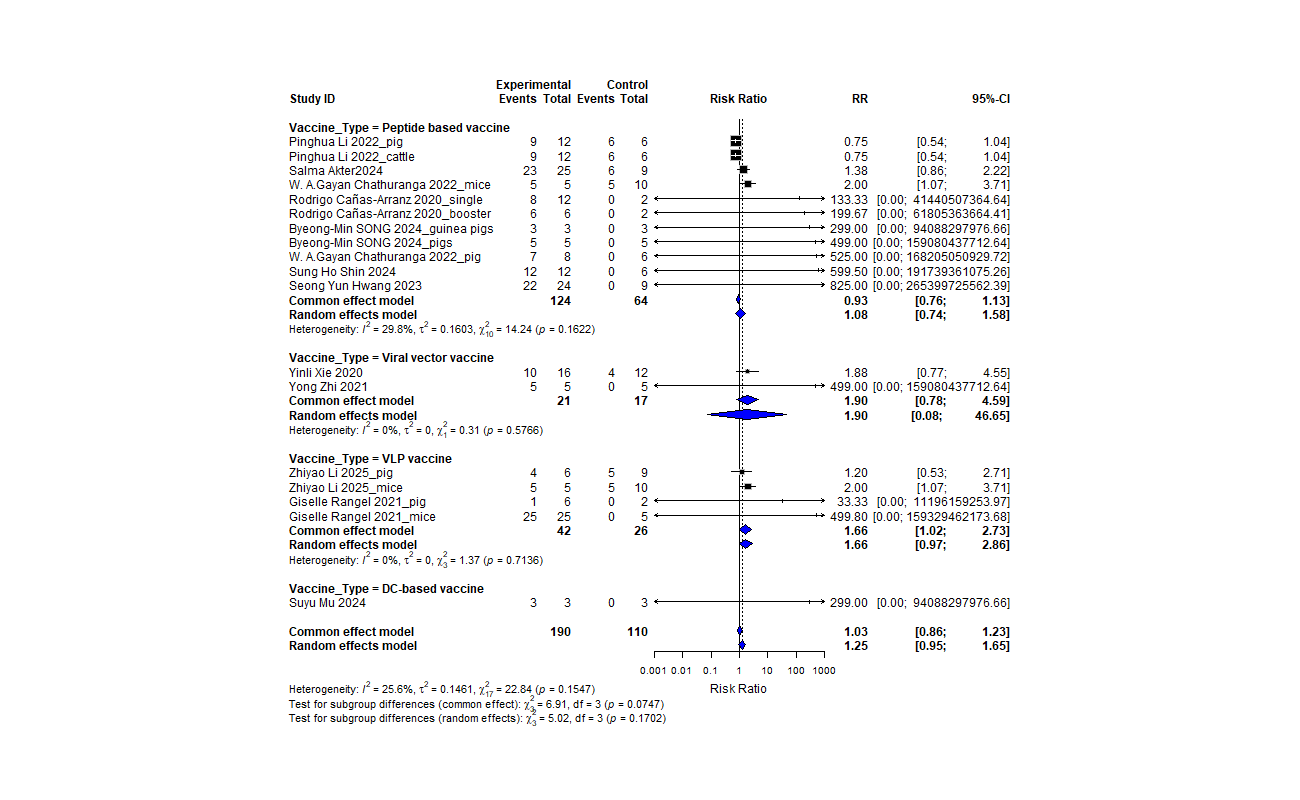


Fig. S12: Forest plot of leave-one-out sensitivity analysis removing one study at a time and recalculating the pooled effect size (Fudong Zhang 2021study removed from 19 total), showing pooled risk ratios (RR) with 95% confidence intervals (CI) for protection outcomes across vaccine platforms using a random-effects model.


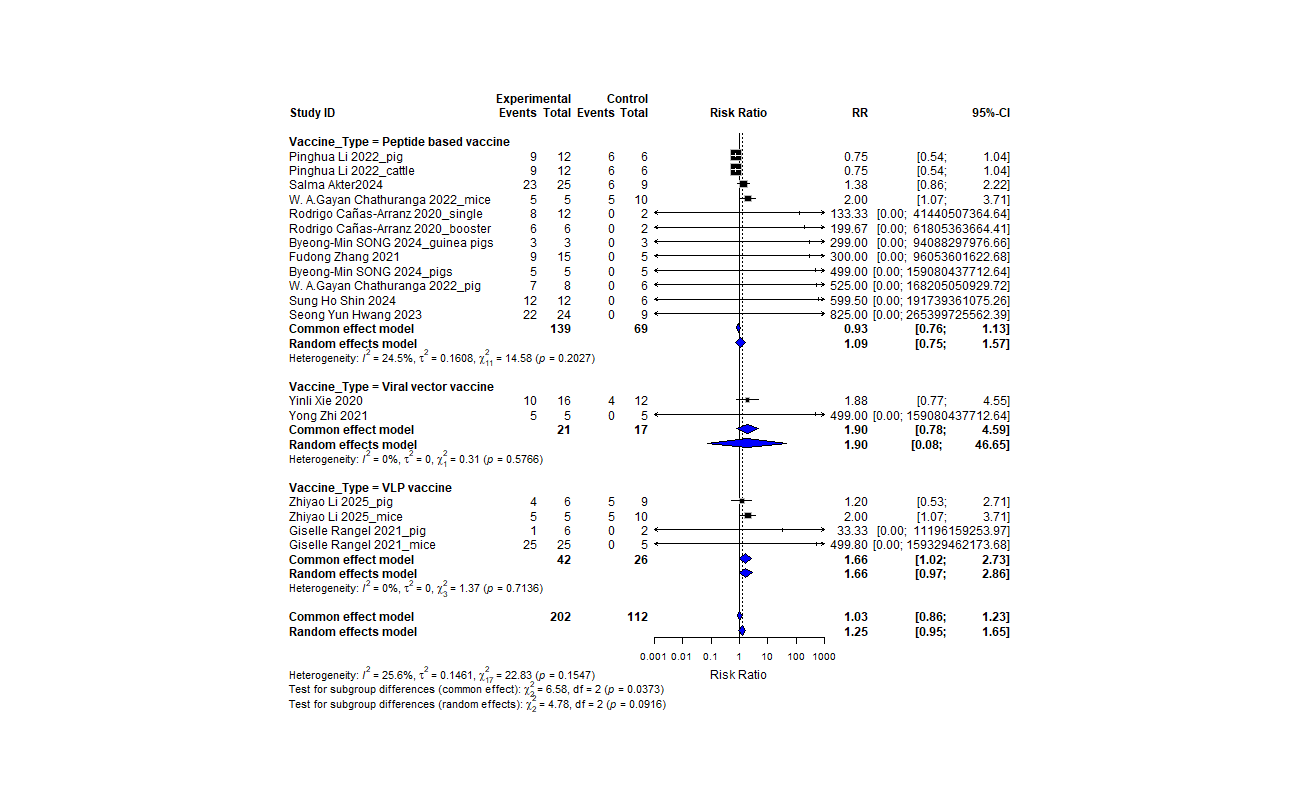


Fig. S13: Forest plot of leave-one-out sensitivity analysis removing one study at a time and recalculating the pooled effect size (Suyu Mu 2024 study removed from 19 total), showing pooled risk ratios (RR) with 95% confidence intervals (CI) for protection outcomes across vaccine platforms using a random-effects model.

The search was conducted across PubMed, Scopus, and Web of Science, covering publications from 2020 to 2025. Exact search strings for each database are provided in Supplementary File 1.

web of science core collection ((((((TI=(foot and mouth disease)) OR TI=(foot-and-mouth disease)) OR TI=(foot-and-mouth disease virus)) OR TI=(FMD)) OR TI=(FMDV)) NOT TI=(hand)) AND TI=(vaccine)

google scholar ((((foot[Title/Abstract] AND mouth disease[Title/Abstract]) OR (foot-and-mouth disease[Title/Abstract])) OR (FMD[Title/Abstract])) AND (vaccine[Title/Abstract])) NOT (hand[Title/Abstract])

scopus ((((foot[Title/Abstract] AND mouth disease[Title/Abstract]) OR (foot-and-mouth disease[Title/Abstract])) OR (FMD[Title/Abstract])) AND (vaccine[Title/Abstract])) NOT (hand[Title/Abstract])

Pubmed ((((foot[Title/Abstract] AND mouth disease[Title/Abstract]) OR (foot-and-mouth disease[Title/Abstract])) OR (FMD[Title/Abstract])) AND (vaccine[Title/Abstract])) NOT (hand[Title/Abstract])

sciencedirect ((((foot[Title/Abstract] AND mouth disease[Title/Abstract]) OR (foot-and-mouth disease[Title/Abstract])) OR (FMD[Title/Abstract])) AND (vaccine[Title/Abstract])) NOT (hand[Title/Abstract])
